# Early risk stratification of late-onset sepsis in very preterm infants by intestinal microbiota profiling: a multicenter case–control validation study

**DOI:** 10.1080/19490976.2026.2693365

**Published:** 2026-07-02

**Authors:** Nina M. Frerichs, Rimke R. de Kroon, Yannick van Schajik, Sofia el Manouni el Hassani, Aranka J. van Wesemael, Willem P. de Boode, Veerle Cossey, Christian V. Hulzebos, Chris H. P. van den Akker, Marlou M. A. Raets, Esther J. d'Haens, Daniel Vijlbrief, Mirjam M. van Weissenbruch, Wouter J. de Jonge, Nanne K. de Boer, Johannes B. van Goudoever, Andrew D. Beggs, Mohammed Nabil Quraishi, Mark Davids, Sudip Mondal, Animesh Acharjee, Hendrik J. Niemarkt, Tim G. J. de Meij

**Affiliations:** a Department of Pediatric Gastroenterology, Emma Children's Hospital, Amsterdam UMC, University of Amsterdam, Amsterdam, the Netherlands; b Amsterdam Reproduction and Development Research Institute, Amsterdam UMC, Amsterdam, the Netherlands; c Amsterdam Gastroenterology Endocrinology Metabolism Research Institute, Amsterdam UMC, Amsterdam, the Netherlands; d Tytgat Institute, University of Amsterdam, Amsterdam UMC, Amsterdam, the Netherlands; e Neonatal Intensive Care Unit, Radboud University Medical Centre, Radboud Institute for Health Sciences, Amalia Children's Hospital, Nijmegen, the Netherlands; f Neonatal Intensive Care Unit, University Hospitals Leuven, Leuven, Belgium; g Neonatal Intensive Care Unit, Beatrix Children's Hospital/University Medical Centre Groningen, Groningen, the Netherlands; h Department of Neonatology, Emma Children's Hospital, Amsterdam UMC, Amsterdam, the Netherlands; i Division of Neonatology, Maastricht University Medical Center+, MosaKids Children's Hospital, Maastricht, the Netherlands; j Neonatal Intensive Care Unit, Amalia Children's Centre, Isala, Zwolle, the Netherlands; k Neonatal Intensive Care Unit, University Medical Centre Utrecht, Utrecht, the Netherlands; l Department of Surgery, University of Bonn, Bonn, Germany; m Department of Gastroenterology and Hepatology, Amsterdam UMC, Amsterdam, the Netherlands; n Department of Cancer and Genomic Sciences, School of Medical Sciences, College of Medicine and Health, University of Birmingham, Birmingham, United Kingdom; o Microbiota Center Amsterdam, Amsterdam, the Netherlands; p Institute of Translational Medicine, University Hospitals Birmingham NHS Foundation Trust, Birmingham, United Kingdom; q MRC Health Data Research UK (HDR UK), Birmingham, United Kingdom; r Neonatal Intensive Care Unit, Máxima Medical Center, Veldhoven, the Netherlands; s Department of Pediatrics, Maastricht University Medical Center, Maastricht, the Netherlands

**Keywords:** Gut microbiome, 16S rRNA gene sequencing, premature neonates, neonatal intensive care unit, bloodstream infection, septicemia

## Abstract

Intestinal bacterial translocation to the bloodstream is a route of infection for late-onset sepsis (LOS) in preterm infants, highlighting the potential of fecal microbiota profiling for early risk stratification. We aimed to identify and validate LOS-specific gut microbiota signatures. Fifty-eight preterm infants (gestational age < 30 weeks) with blood culture-proven LOS (excluding coagulase-negative *staphylococci*) were matched to controls (1:1) across three cohorts (Discovery (DC) *n* = 18; Validation 1 and 2; VC1 *n* = 12, VC2 *n* = 28). Fecal samples collected up to 10 days before LOS onset underwent 16S rRNA gene sequencing. Microbial composition, diversity, and discriminatory taxa were compared across LOS subgroups. Random Forest (RF) models were trained in DC and validated in VC1/VC2. Microbiota variation was largely explained by LOS pathogen (R^2^ = 17%, *P* < 0.001). Infants with non-staphylococcal and *E. coli*-LOS showed a temporal increase in relative abundance of *Escherichia/Shigella*. The RF model distinguishing *E. coli-*LOS from controls displayed the highest discriminatory performance (AUC = 0.99/0.78/0.61 for DC/VC1/VC2) compared to non-staphylococcal LOS (AUC = 0.96/0.46/0.41). Our findings demonstrate profound microbiota shifts preceding *E. coli*–LOS, with higher discriminatory ability compared to non-staphylococcal-LOS. While pathogen-specific microbiota-based risk stratification may offer added clinical value, reduced validation performance highlights the limited generalizability and underscores the need for future research before clinical translation.

## Introduction

Late-onset sepsis (LOS) is a major cause of morbidity and mortality among preterm infants.^1^ Depending on gestational age (GA) and birth weight (BW), reported incidence varies between 10% and 40%.^2^ ^,^ ^3^ Accurate and timely LOS diagnosis is essential to mitigate negative short- and long-term health outcomes. However, diagnosing LOS is often challenging due to the non-specific clinical symptoms at disease onset,^4‐6^ and therefore poses a clinical dilemma: delaying treatment increases the risk of morbidity and mortality, whereas initiating empirical antibiotic therapy without diagnostic certainty contributes to antimicrobial resistance. Additionally, disruption of the neonatal microbiome as a result of antibiotics has been linked to unfavorable health outcomes.^7^ ^,^ ^8^

Blood culture is the golden standard for LOS diagnosis, but is hampered by a long turn-around time (36–48 hours).^9^ Additionally, blood cultures are prone to contamination and have a suboptimal sensitivity, particularly when low volumes of blood are used.^10^ ^,^ ^11^ Novel diagnostic markers (e.g., interleukin-6, presepsin) and continuous monitoring of heart rate variability may serve as adjuvant diagnostic biomarkers for LOS, but lack specificity.^5^ ^,^ ^8^ ^,^ ^12^ ^,^ ^13^ Taken together, there is an urgent need for noninvasive, accurate, preclinical diagnostic biomarkers for LOS in the preterm population, to enable timely initiation of appropriate treatment.^8^

Fecal microbiota–based biomarkers have been hypothesized as a promising tool for the early diagnosis of LOS. In a subset of LOS episodes, predominantly caused by Gram-negative bacteria the LOS-causing pathogen colonizes the preterm intestinal tract and subsequently translocates into the bloodstream^14^ Earlier studies have reported profound alterations in the gut microbiota composition and microbial metabolites preceding LOS onset.^7,14‐17^ Several days before onset of LOS, an increase in the fecal relative abundances (RA) of opportunistic pathogens, specifically Gram-negative bacteria, was demonstrated. High genetic similarity of these pathogens, as confirmed though shotgun sequencing, was demonstrated between fecal and blood isolates of LOS-affected infants.^7^ ^,^ ^14^ ^,^ ^15^

These findings illustrate the potential of intestinal microbiota profiling for early gut-derived LOS risk stratification. Yet, most studies lacked consistent replication or validation in external cohorts, and showed considerable heterogeneity in the timing of sampling and microbiome profiling methods, limiting cross-study comparability. Moreover, stratification by causative pathogen or presumed route of infection (gut-derived vs. catheter-associated) is not frequently done, potentially resulting in the loss of important pathophysiological information. The longitudinal trajectory of the gut microbiota prior to LOS also remains poorly understood. To address these gaps, we conducted a longitudinal prospective multicenter case‒control study in preterm infants (GA < 30 weeks). We aimed to (1) identify microbiota differences up to 10 days before LOS onset, (2) develop a microbiota-based machine learning (ML) model for early LOS risk stratification in a Discovery Cohort, and (3) validate the identified microbiota differences and model in two independent validation cohorts.

## Methods

### Study design

The study was embedded in a multicenter prospective study aimed at the development of noninvasive fecal biomarkers for LOS and necrotizing enterocolitis (NEC). For this purpose, fecal samples and clinical data are collected daily from all live-born preterm infants (GA <  30 weeks) up to 29 days after birth at nine neonatal intensive care units (NICUs) in the Netherlands and Belgium. Infants with congenital gastrointestinal abnormalities or chromosomal disorders were excluded. Ethical approval was provided by the local medical ethical committees of all participating centers (2014.386 (A2020.190)). Written informed consent was obtained from parents or legal caretakers.

### Cohort description

**Figure 1.**
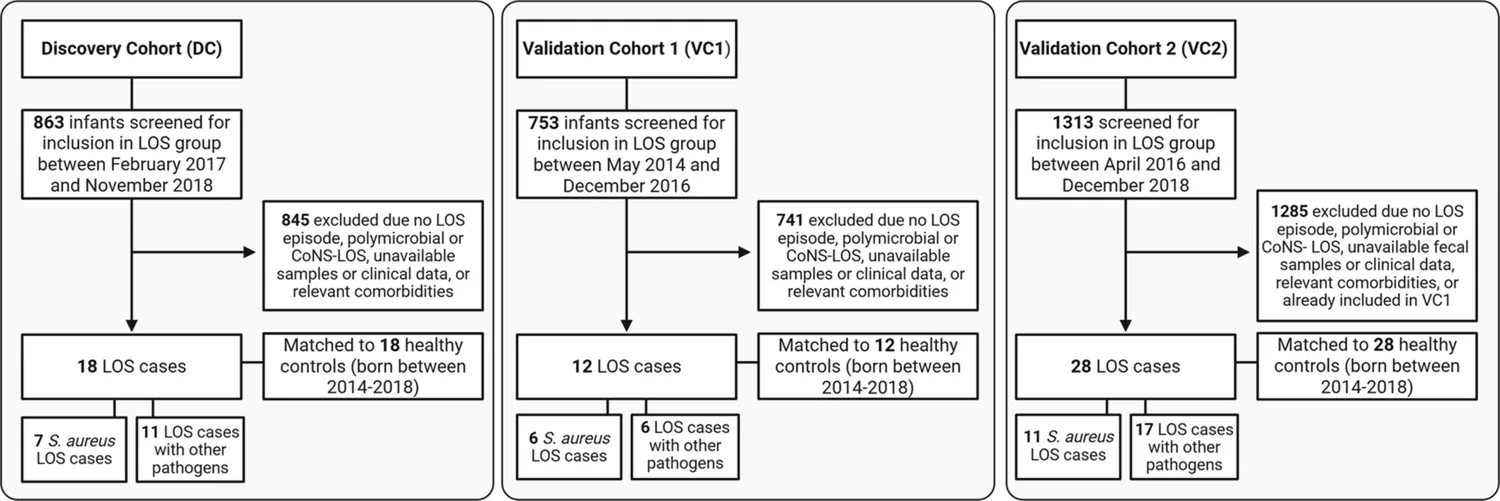
Flowchart displaying the inclusion process in Discovery Cohort (DC), Validation Cohort (VC1), and Validation Cohort 2 (VC2). *Created in BioRender. Amsterdamumc, Eminds. (2026)* https://BioRender.com/xvlxzz6.

The current study consists of three unique case‒control cohorts: the Discovery Cohort (DC) and two validation cohorts (VC1 and VC2). DC, VC1, and VC2 were all compiled from the overarching prospective cohort study, covering different time periods (Figure 1). The DC was the first dataset available for analysis and provided the initial basis for identifying the microbial signal associated with LOS. For the DC, LOS-affected infants born between February 2017 and November 2018 were included (Figure 1). Subsequently, we aimed to validate the findings from the DC in two independent validation cohorts. To achieve this, we utilized a previously published cohort^15^ of which residual DNA isolates were available, referred to as VC1, with LOS-affected infants born between May 2014 and December 2016 and a pathogen distribution similar to the DC (Figure 1, Table 1). In VC1, samples were analyzed up to 5 days before diagnosis. VC2 was compiled specifically for validation of the DC, including affected infants born between April 2016 and December 2018 with samples up to 10 days before diagnosis (similar to DC) and a broader range of LOS pathogens (Figure 1, Table 1). Analyzing the three cohorts across different time periods enables the identification of robust microbiota-based LOS signals. Some temporal overlap exist between DC and VC1, as additional infants met the study criteria as more data became available over time. To prevent duplication, we confirmed that all VC2 infants were distinct from those in DC using unique identifiers and database cross-checking.

**Table 1.** Overview of included late-onset sepsis cases per cohort.

Blood culture-isolated pathogen	Discovery Cohort samples (cases)	Validation Cohort 1 samples (cases)	Validation Cohort 2 samples (cases)	Total samples (cases)
*Staphylococcus aureus*	34 (7)	15 (5)	39 (11)	89 (23)
Non-staphylococcal LOS				
*Escherichia coli*	26 (4)	13 (5)	14 (5)	53 (14)
*Enterobacter aerogenes*	n.a.	n.a.	5 (1)	5 (1)
*Enterobacter cloacae*	11 (2)	n.a.	19 (4)	30 (6)
*Enterococcus faecalis*	6 (2)	5 (1)	3 (1)	14 (4)
*Klebsiella pneumoniae*	19 (2)	4 (1)	n.a.	19 (3)
*Klebsiella oxytoca*	5 (1)	n.a.	4 (1)	9 (2)
Group B *streptococcus*	n.a.	n.a.	19 (2)	19 (2)
*Serratia marcescens*	n.a.	n.a.	9 (3)	9 (3)
Total LOS samples (cases) per cohort	101 (18)	37 (12)	112 (28)	250 (58)

### Selection and matching criteria

No infants (cases or controls) with blood culture-proven early-onset sepsis, NEC (Bell's stage 2 A or higher), and/or focal intestinal perforation in the first 29 days of life were included.

LOS-affected infants were included if they fulfilled the Vermont Oxford Network criteria for LOS: (1) clinical signs of a generalized infection, (2) a positive blood culture taken ≥72 hours after birth, and (3) the start of antibiotic treatment with the intention to treat for ≥5 days. Only the first episode of LOS was included. LOS caused by coagulase-negative *staphylococci* (CoNS), defined as a positive CoNS-blood culture with a C-reactive protein (CRP) level > 10 mg/L surrounding onset of generalized signs of infection,^18^ was excluded to prevent overrepresentation of this subgroup that is generally catheter-associated.^19^ LOS onset (t = 0) is defined as the day on which the diagnostic work-up was conducted resulting in LOS diagnosis, including at least a blood culture.

Each LOS-affected infant was matched to a control infant without LOS (1:1). The cohort-specific matching criteria are defined in Appendix A and Table 2. In brief, infants in DC and VC2 were matched based on center of birth, gestational age (±5 days), birth weight (±150 gr), and postnatal age at t = 0 to ensure alignment of fecal sampling window. Infants in VC2 were solely matched based on the center of birth, gestational age (±2 days), and postnatal age at t = 0.

**Table 2.** Overview of matching criteria, sample selection, sample preparation, DNA extraction, gene amplification, and library preparation and sequencing for each cohort.

	Matching criteria	Sample selection	DNA extraction	Gene amplification	Library preparation and sequencing
Discovery Cohort	Center of birth, GA (± 5 days), BW (± 150 gr), and PNA at LOS onset (± 0 day)	≥2 fecal samples per infant, t-1 to t-10	QIAamp PowerFecal DNA Kit (Qiagen, Hilden, Germany)	Earth Microbiome Project Protocol	Paired-end sequenced (2 × 250 bp) on Illumina MiSeq platform
Validation Cohort 1	Center of birth, GA (± 2 days), PNA at LOS onset (± 0 day)	≥2 fecal samples per infant, t-1 to t-5	Easy Mag extraction kit (Biomérieux, Marcy l’Etoile, France)		
Validation Cohort 2	Center of birth, GA (± 5 days), BW (± 150 gr), and PNA at LOS onset (± 0 day)	≥2 fecal samples per infant, t-1 to t-10	PSP Spin Stool DNA Plus Kit (Invitek Molecular/Isogen Life Science, De Meern, the Netherlands)	Previously described by Kozich *et al.* (2013).	

Abbreviations: BW, birthweight; d, days; GA, gestational age; gr, grams; LOS, late-onset sepsis; PNA, postnatal age; t-1, 1 days before LOS (and so forth)

### Sample and data collection

Fecal samples and clinical data were collected daily during the first 29 days of life. Fecal samples were collected from diapers into sterile containers by NICU nurses trained according to a standardized protocol across cohorts to ensure consistent sample collection. Samples were stored at −20 °C, reflecting standard practice during the study period in the participating centers, as large-scale sampling storage at -80°C was not available at the time. Both sample handling practices and storage conditions were similar across cohorts. Table S1 displays clinical data extracted from the electronic patient files.

### Sample analysis

The sample selection, sample preparation, DNA extraction, gene amplification, library preparation, and 16S rRNA gene sequencing are summarized in Table 2 and described in detail in Appendix A. Differences exist in the wet-lab analytical pipelines (DNA extraction and gene amplification methods), driven by changes in laboratory workflows over time (for DC and VC2) and the pre-existing nature of VC1. The bioinformatic workflow was harmonized across all cohorts. The sequencing depth per cohort is displayed in Table S2. The number of included samples per timepoint for both LOS-affected infants and controls, and the number of cases for each pathogen can be found in Table 1 and S3, respectively.

### Data processing

Data processing was the same for the three cohorts. The amplicon sequences were paired using a pipeline based on VSEARCH (v2.15.2).^20^ Paired-end reads were merged, allowing for staggered overlap and a maximum of 100 mismatches. Amplicon sequence variants (ASVs) were inferred from reads with an expected error rate <1.5 using the cluster_unoise algorithm with centroids, requiring a minimum abundance of 4. Chimeric sequences were removed using the uchime3 denovo method. For each sample, ASV abundances were determined by mapping the merged reads against ASV sequences using usearch_global with a 0.97 distance cut-off. Taxonomy was assigned using R (4.2.0), the dada2 assign taxonomy function, and the SILVA 3 (v.132) reference database.^21^ ^,^ ^22^ A phylogenetic tree was generated with MAFFT (v7.310) and FastTree 2 (2.1.11).^23^ ^,^ ^24^

### Statistical analysis

#### Demographics

The demographic and clinical data were analyzed using IBM SPSS Statistics version 28 (SPSS Inc. Chicago). Where appropriate, the Chi-Square test, Fisher's exact test, or a Mann–Whitney U test was used to calculate *P*-values. A *P-*value < 0.05 was considered statistically significant.

#### Microbiota analyses

**Figure 2.**
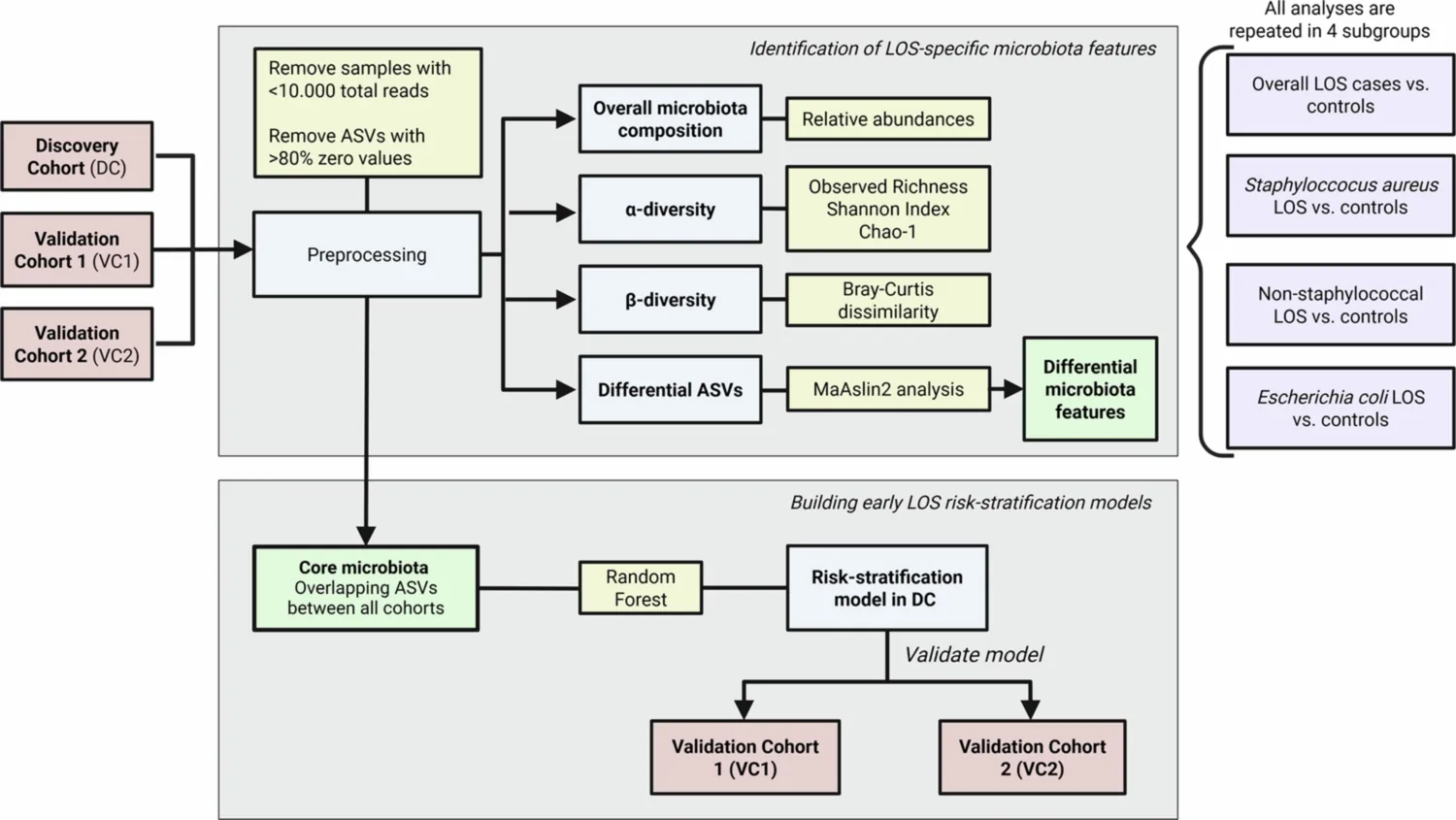
Flowchart displaying the workflow of the microbiota analysis and development of the late-onset sepsis risk stratification models in the Discovery Cohort and Validation Cohort 1 and 2. *Created in BioRender. Amsterdamumc, Eminds (2026)* https://BioRender.com/6s935is.

The microbiota analyses were done in R-studio (version 4.4.3). All analyses were first performed in the DC and repeated in VC1 and VC2 to evaluate the reproducibility of findings (Figure 2).

- The analyses were performed in four (sub)groups:
  1. Overall LOS group (all pathogens combined) vs. controls

- Subgroups of overall LOS, based on the presumed route of infection:
  2. Catheter-derived: *Staphylococcus aureus* (*S. aureus*)-LOS vs. controls
  3. Gut-derived: non-staphylococcal LOS (all other pathogens, excluding *S. aureus*) vs. controls

- Pathogen-specific subgroup of the non-staphylococcal LOS group:
  4. *Escherichia coli* (*E. coli*)-LOS vs. controls

*E. coli* was the only pathogen present in all three cohorts with a presumed gut-derived etiology^14^ and thus the only individual pathogen that was compared across all cohorts.

*Data filtering*: ASVs assigned to nonbacterial domains were removed using the Phyloseq package. Low-depth samples with <10.000 total reads were removed from the dataset,^25^ followed by removal of ASVs with >80% zero values.^26^ Prior to *α*- and *β*-diversity, and MaAslin2 analysis, rarefaction was applied.

*α-diversity:* *α*-diversity on ASV level was calculated using the observed richness, Shannon index, and Chao1 index.^27^ Significant differences between disease subgroups and timepoints were determined by Kruskal–Wallis analysis.^28^ Additionally, *α*-diversity was assessed using linear mixed-effects models (LMM) to account for repeated measurements within infants. Disease status, time period (1–3 days, 4–6 days, 7–8 days, and 9–10 days before LOS, respectively), and their interaction were included as fixed effects, with patient ID included as a random intercept. Model-adjusted means and 95% confidence intervals were estimated using marginal means. A *P*-value < 0.05 was deemed significant.

*β-diversity*: Count data were converted to RAs within each sample. Bray–Curtis dissimilarities were subsequently calculated based on these normalized data and visualized using Principal Coordinates Analysis (PCoA). Permutational multivariate analysis of variance (PERMANOVA) tests were conducted corrected for patient ID.^29^ The homogeneity of dispersion was checked for each group to ensure PERMANOVA assumptions were met. A *P*-value < 0.05 was deemed significant. Additionally, time-stratified *β*-diversity analysis was conducted. Samples were grouped into four temporal windows based on days relative to LOS onset. Within each window, only one sample per patient was retained (sample closest to LOS) to avoid over-representation and ensure balanced longitudinal comparison.

*Taxonomic composition:* Total-sum scaling was used to calculate RA per taxa per sample. Taxa were assigned to the lowest available taxonomic level (species, genus, family, order, class, or phylum). Missing taxonomic labels were set to *Unclassified*. The RA for the subgroup comparisons were calculated as mean per-sample RA within each subgroup and time period (1–3 days, 4–6 days, 7–8 days, and 9–10 days before LOS). The top 25 taxa were selected by ranking mean per-sample RA across all samples per cohort. Remaining taxa were grouped as *Other*. To avoid overinterpretation of species-level assignments, *Staphylococcus* ASVs were collapsed and reported as *Staphylococcus* species. Stacked bar charts were computed for each comparison and time period to assess longitudinal changes in the days preceding LOS onset in cases versus controls. Boxplots were created to visualize the distribution of sample-level RA for the three taxa with the highest mean RA per cohort. Additionally, LMM were used to assess longitudinal differences in RA of three top taxa between groups. RA was averaged per patient within each predefined time period (1–3 days 4–6 days, 7–8 days, and 9–10 days before LOS). Disease status (case/control), time period (categorical), and their interaction were included as fixed effects, with patient ID as a random intercept. Model-adjusted means and 95% confidence intervals were estimated using marginal means. A *P*-value < 0.05 was deemed significant.

*MaAsLin2:* multivariable associations between disease status and microbiota (ASV level) were assessed using microbiome multivariable association with linear models (MaAsLin2) for all subgroups. RAs were obtained through total-sum scaling on the sample level, and log-transformed prior to analysis. Disease status and time periods were included as fixed effects, while patient ID was used as a random effect to account for within-subject correlations. The differentiating features are represented by two or more annotated ASVs with *q*-value < 0.05 (adjusted *P*-value).

*Machine learning:* Two separate microbiota-based risk-stratification models were build using ML for discrimination of non-staphylococcal LOS from controls (1) and *E. coli*-LOS from controls (2), trained on the core microbiota data, defined as the overlapping ASVs between the three cohorts. First, filtering was applied to retain the common OTUs across all cohorts. Next, zero-variance and low-informative features were removed to reduce sparsity and improve model stability. To appropriately handle zero values in compositional data, a small pseudo-count (+1) was added to all counts prior to transformation. Microbial abundances were subsequently transformed using the centered log-ratio (CLR) transformation.^30^ ^,^ ^31^ Finally, CLR-transformed features were z-normalized prior to modeling.

Random Forest (RF) classifiers were trained using repeated cross-validation. For non-staphylococcal LOS, the dataset was randomly divided into training (80%) and test (20%) subsets, and model training employed 10-fold cross-validation repeated three times. For *E. coli*-LOS, a more stringent modeling strategy was applied to account for the limited sample number. Here, repeated 3-fold cross-validation was used with 5 repeats. This approach ensured each fold contained a representative number of cases, improving reliability and reducing overfitting in small sample conditions. Standard RF classifiers treat samples as independent observations and do not account for within-subject correlation in repeated-measures designs. Therefore, models were trained exclusively in the DC and evaluated in two independent validation cohorts with no subject overlap. Subject-level data splitting was applied to ensure that all samples from the same infant were assigned to either the training or test set (grouped cross-validation), reducing the potential for within-subject information leakage. Variable importance scores were calculated using the ranger RF algorithm (mean decrease in Gini index) to quantify each taxon's relative contribution to model performance. The final trained models were applied to VC1 and VC2 to assess generalizability. Predictive performance was evaluated using the receiver operating characteristic curve (ROC–AUC), sensitivity, specificity, and F1-score for each cohort.

To further assess the model performance, precision–recall (PR) curve analysis was conducted using cross-validated predictions from the RF model. PR curves were generated using the PRROC package by plotting precision against recall across varying decision thresholds. The Area Under the PR Curve (PR-AUC) was calculated as a summary metric of the model performance, with the baseline defined as the prevalence of the positive class in the dataset. PR-AUC was reported alongside ROC-AUC to provide a more informative evaluation of classifier performance for the minority class.

*Confounding parameters*: the impact of potential confounding parameters on *α*- and *β*-diversity, calculated as described above, was assessed across cohorts. Parameters included birth weight, gestational age, delivery mode, feeding type, age at time of sampling, antibiotic exposure prior to t = 0, presence of invasive medical devices in 48 hours prior to t = 0, and fecal sample storage duration prior to microbiota analysis. *α*-diversity associations were evaluated using LMM, with patient ID as a random effect and disease state as a covariate when applicable. *β*-diversity associations were tested using PERMANOVA with 999 permutations. All models were fitted per cohort and *P*-values were adjusted using False Discovery Rate (FDR) correction. Effect sizes with 95% confidence intervals were reported for *α*-diversity, and R²-based heatmaps were used to visualize *β*-diversity associations.

## Results

### Cohort characteristics

58 LOS cases and 58 matched controls were selected and analyzed: 18 cases (101 samples) and 18 controls (115 samples) in the DC, 12 cases (37 samples) and 12 controls (33 samples) in VC1, and 28 cases (112 samples) and 28 controls (139 samples) in VC2 (Table 1 and S3). Table S4 shows the data filtering procedures and sample dropouts per cohort. The clinical characteristics for all infants are presented in Table 1. No significant differences were observed for the majority of characteristics (Table 3). Notably, the postnatal age of onset of LOS did differ significantly between cohorts (median day [Q1, Q3]: DC = 15 [12–20], VC1 = 13 [6–17], VC2 = 12 [9–17], *p* = 0.022). The separate baseline characteristics for DC, VC1, and VC2 can be found in Tables S5–S7, respectively.

**Table 3.** Cohort characteristics comparing the Discovery Cohort, Validation Cohort 1, and Validation Cohort 2.

	DC (*n* = 36)	VC1 (*n* = 24)	VC2 (*n* = 28)	*P*-value
**Baseline demographic characteristics**				
Gestational age (days), median [Q1–Q3]	26 + 3 [25 + 2 − 28 + 3]	27 + 3 [26 + 3 − 27 + 4]	26 + 4 [25 + 2 − 28 + 5]	0.245
Birth weight (grams), median [Q1–Q3]	905 [733–1073]	1040 [800–1146]	853 [710–1024]	0.260
Biological sex (Female, *n* [%])	15 [42]	12 [50]	30 [54]	0.535
Singleton, *n* [%]	26 [72]	17 [71]	31 [55]	0.188
Apgar score 5 min, median [Q1–Q3]	7 [6–8]	7 [6–8]	8 [6–9]	0.649
Mode of delivery (Vaginal *n* [%])	12 [33]	12 [50]	24 [43]	0.418
Mortality (Yes, *n* [%])	1 [3]	2 [8]	4 [7]	0.601
Length of NICU stay (days), median [Q1–Q3]	49 [31–76]	29 [22–48]	48 [28–63]	**0.043**
Day of life blood-culture confirmed sepsis (t = 0), median [Q1–Q3]	15 [12–19]	12.5 [6–17]	12 [9–17]	**0.022**
**Medication practices**				
Ratio of antibiotic administration in first 29 days of life^1^, median [Q1–Q3]	0.45 [0.25–0.62]	0.25 [0.13–0.60]	0.50 [0.25–0.70]	0.078
Ratio of antibiotic administration before t = 0^2^, median [Q1-Q3]	0.25 [0.19–0.45]	0.27 [0.02–0.50]	0.43 [0.24–0.61]	**0.026**
Exposure to antibiotics prior to t = 0 (Yes, *n* [%])	32 [89]	18 [75]	52 [93]	0.078
Surfactant administration (first 4 lifedays), *n* [%]				
None	6 [18]	1 [5]	5 [10]	0.597
1× surfactant administration	9 [27]	5 [24]	14 [26]	
2× or more surfactant administration	19 [56]	15 [71]	34 [64]	
Antenatal corticosteroids, *n* [%]				
None	13 [36]	11 [48]	19 [34]	0.819
Incomplete course	12 [33]	7 [30]	21 [37]	
Complete	11 [31]	5 [22]	16 [29]	
**F** **eeding practices**				
Full enteral feeding lifeday, median [Q1–Q3]	10.5 [8–15]	9 [8–12]	10 [9–13]	0.399
Feeding type at full enteral feeding lifeday^3^, *n* [%]				
Exclusively human milk^4^	21 [70]	13 [62]	33 [72]	0.153
Exclusively formula feeding	0 [0]	4 [19]	4 [9]	
Mix of human milk and formula feeding	9 [30]	4 [19]	9 [20]	
Formula feeding in the first 29 days (Yes *n* [%])	21 [70]	19 [86]	43 [83]	0.266
Reached full enteral feeding at t = 0 (Yes, *n* [%])	25 [78]	11 [50]	33 [66]	0.099
Parenteral feeding before t = 0 (days), median [Q1–Q3]	10.5 [8–13]	8 [5–11]	10 [8–12]	0.066
**Exposure to invasive medical devices**				
Exposure to invasive medical device (48 hours prior to t = 0) (Yes, *n*[%])	26 [72]	16 [67]	48 [86]	0.113
Peripheral IV (Yes, *n*[%])	23 [64]	12 [50]	36 [64]	0.449
Central catheter (Yes, *n*[%])	10 [28]	6 [25]	20 [36]	0.560
Invasive ventilation (Yes, *n*[%])	9 [25]	1 [4]	19 [34]	**0.019**
Invasive ventilation before t = 0 (days), median [Q1–Q3]	1 [0–5]	1 [0–3]	2 [0–8]	0.082

^1^ The ratio of antibiotic administration was calculated as the number of days with documented antibiotic use divided by 29, representing the proportion of the first 29 days exposed to antibiotics.

^2^ The day on which the diagnostic work-up resulting in the LOS diagnosis was performed (and the corresponding matched day for the control infant) was defined as t = 0.

^3^ Full enteral feeding was defined as the first day on which both parenteral nutrition and intravenous glucose were ceased.

^4^ Human milk was defined as mother's own milk, donor human milk, or a combination. Missing data: surfactant administration: n = 2 in DC, n = 3 in VC1, and n = 3 in VC2; antenatal corticosteroids: n = 1 in VC1; full enteral feeding lifeday: n = 4 in DC, n = 3 in VC1, and n = 6 in VC2; feeding type at full enteral feeding lifeday: n = 6 in DC, n = 3 in VC1, and n = 10 in VC2; formula feeding in first 29 days of life: n = 6 in DC, n = 2 in VC1, and n = 4 in VC2; reached full enteral feeding at t = 0: n = 4 in DC, n = 2 in VC1, and n = 6 in VC2; parenteral feeding before t = 0: n = 2 in DC and n = 1 in VC1. Abbreviation: NICU = neonatal intensive care unit.

### Lower *α*-diversity and increased fecal sample clustering based on blood culture-isolated pathogens in the Discovery Cohort

**Figure 3.**
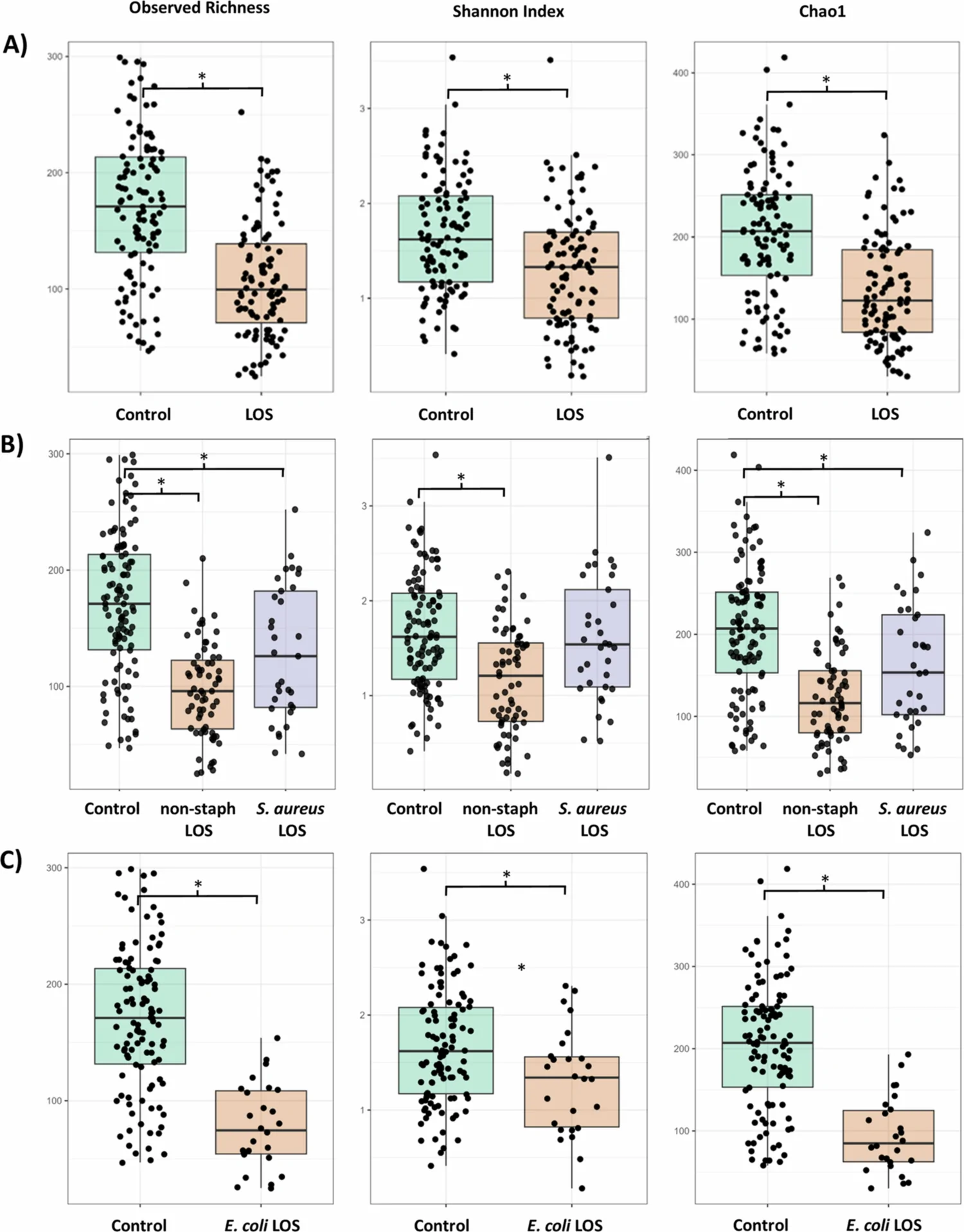
*α*-diversity in fecal samples up to 10 days prior to clinical onset of late-onset sepsis versus controls in the Discovery Cohort. The boxplots display the distribution of three *α*-diversity metrics (Richness, Shannon index, and Chao1, respectively) for A) infants with LOS versus controls (*P* < 0.05), B) infants with *Staphylococcus aureus* LOS, non-staphylococcal LOS, and controls (*P* < 0.05), and C) *E. coli*-LOS versus controls (*P* < 0.05). This figure demonstrates a decrease in *α*-diversity in preterm infants with LOS compared to controls in the Discovery Cohort. A *P*-value < 0.05, as assessed by a Wilcoxon Rank-Sum or Kruskal‒Wallis test, was considered significant. LOS, late-onset sepsis; non-staph, non-staphylococcal.

In the DC, across all *α*-diversity metrics, LOS-affected infants displayed a lower *α*-diversity compared to controls, both for the overall LOS group and within each subgroup (*P* < 0.01, Wilcoxon Rank-Sum U) (Figure 3). The largest difference was seen for *E. coli*-LOS vs. controls. This pattern persisted longitudinally across subgroups, showing consistently lower *α*-diversity in cases compared to controls over time (*P* < 0.05) (Figure S1). Additionally, LMM were applied to account for repeated measurements per infant over time, and also revealed, although less pronounced, significant differences in *α*-diversity between the subgroups with most extensive differences between non-staphylococcal LOS vs. controls and *E. coli*-LOS vs. controls (Table S8). We additionally assessed associations between clinical factors and *α*-diversity indices. Apart from a significant association with disease state, no other clinical factors were significantly associated with *α*-diversity (Figure S2).

**Figure 4.**
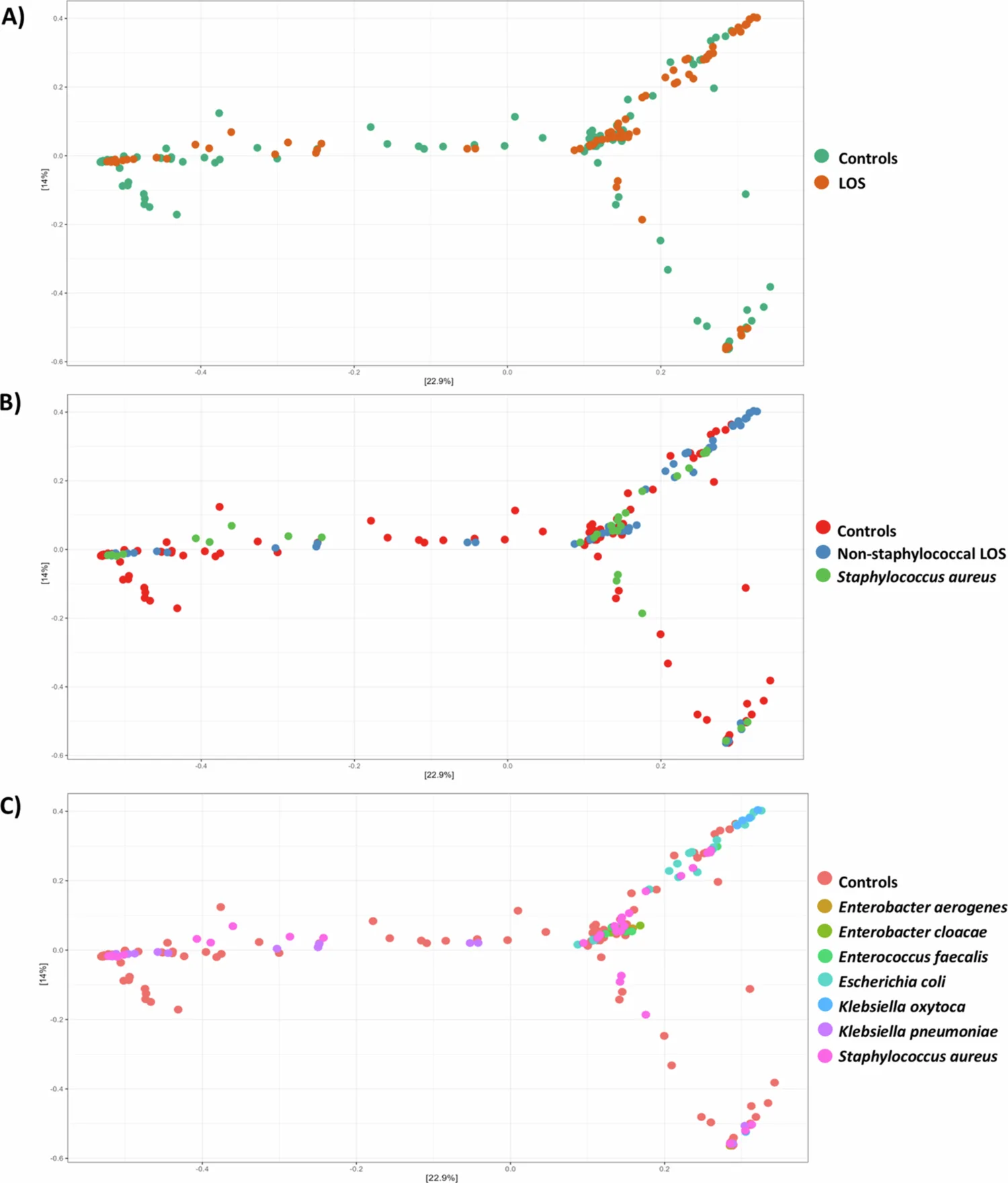
*β*-diversity in fecal samples up to 10 days prior to clinical onset of late-onset sepsis versus controls for the Discovery Cohort. The bacterial *β*-diversity as assessed by PCoA based on Bray‒Curtis dissimilarity is displayed. Potential clustering of fecal samples based on microbiome composition is assessed in all infants with LOS versus controls (A), *S. aureus*-LOS, non-staphylococcal LOS, and controls (B), and LOS by specific causative pathogens (pathogens isolated from the blood culture) versus controls (C). All data were normalized before assessment. Statistical analysis was performed by PERMANOVA. A *P*-value below 0.05 was considered significant. This figure demonstrates that, in DC, the largest variation in *β*-diversity is explained by blood culture isolated pathogen (C, R^2^ = 17.2%, F = 6.0, *P* = 0.001), followed by type of sepsis (B, R^2^ = 5.3%, F = 5.8, *P* = 0.001), and least variation is explained by disease state (LOS vs. controls, A, R^2^ = 3.6%, F = 7.9, *P* = 0.001).

*β*-diversity clustering was assessed by PCoA based on Bray–Curtis dissimilarities. Clustering was primarily explained by patient ID (R^2^ = 53%, F = 5.6, *P* = 0.001). Among LOS discriminatory factors, stratification by individual blood culture-isolated pathogens explained the majority of variation (R^2^ = 17.2%, F = 6.0, *P* = 0.001) (Figure 4C), followed by the (sub)group divisions (*S. aureus* vs. non-staphylococcal LOS vs. controls; R^2^ = 5.3%, F = 5.8, *P* = 0.001 (Figure 4B), and overall LOS vs. controls; R^2^ = 3.6%, F = 7.9, *P* = 0.001 (Figure 4A). *β*-diversity was additionally assessed longitudinally using one sample per patient per time bin to account for repeated measures. Longitudinal patterns mirrored pooled *β*-diversity with greater variation after subgroup stratification (overall LOS vs. controls: R^2^ = 3% to 9%, *S. aureus* vs. non-staphylococcal LOS vs. controls: R^2^ = 5% to 14%, individual blood culture-isolated pathogens vs. controls: R^2^ = 14% to 30%) (Figure S3). Lastly, we assessed associations between clinical parameters and *β*-diversity, which explained limited variance, with LOS-associated factors as the main drivers, alongside modest effects of feeding type and center of birth (Figure S4).

### Distinct fecal microbiota composition prior to non-staphylococcal LOS and *E. coli*-LOS in the Discovery Cohort

**Figure 5.**
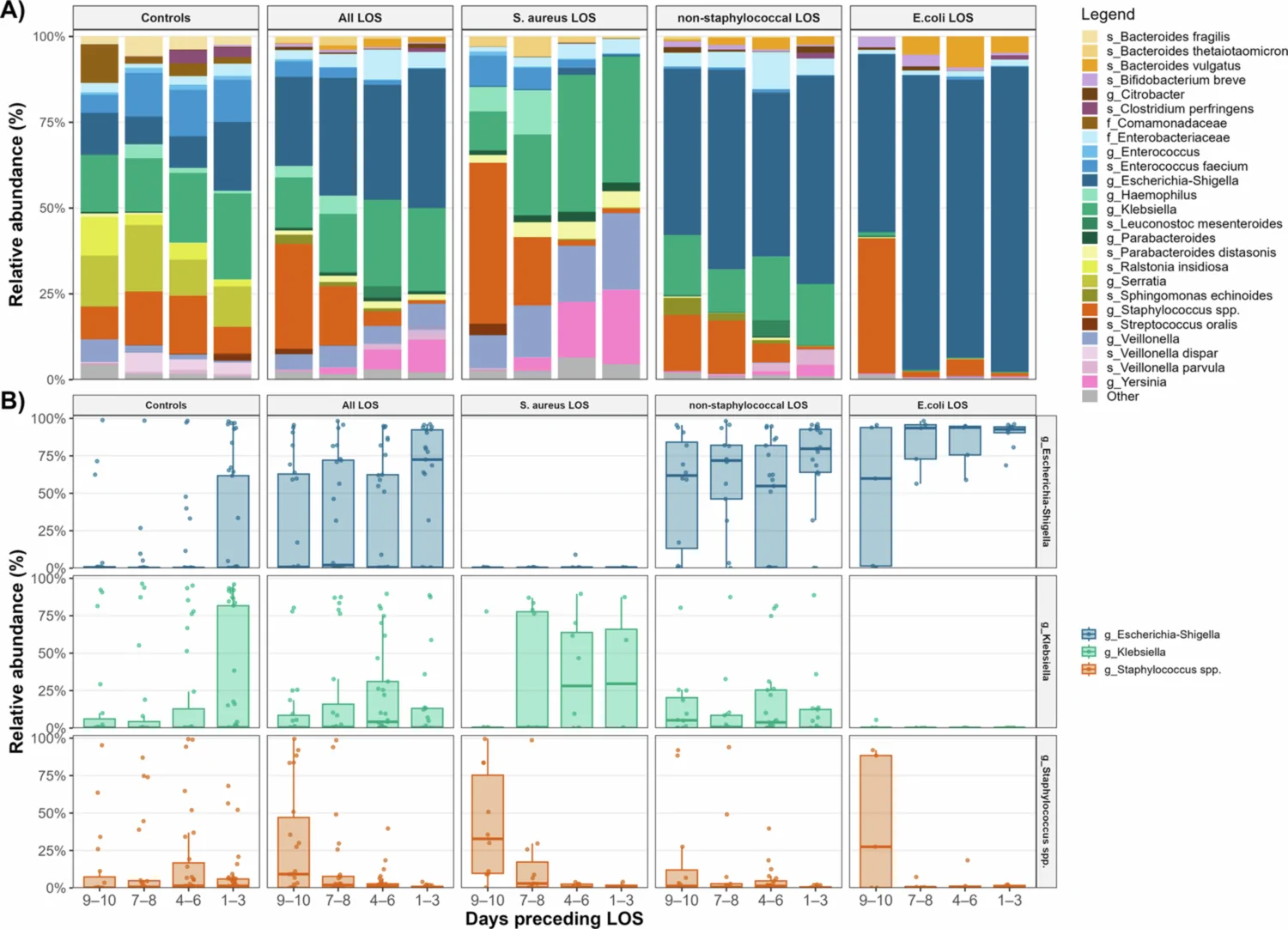
Temporal dynamics of gut microbiota composition at the genus level during the 10 days window preceding clinical onset of late-onset sepsis (LOS) in infants and controls. A) The figure presents stacked bar charts illustrating the relative abundance (RA) (Y-axis, %) of the 25 predominant bacterial genera across defined time intervals preceding LOS onset or the corresponding t = 0 of the control infants (X-axis: 1–3 days, 4–6 days, 7–8 days, and 9–10 days before LOS). RAs were averaged per individual within each time interval. From left to right, panels depict the microbial composition of control infants, infants with LOS caused by any pathogen (all LOS), infants with *Staphylococcus aureus* LOS, infants with non-staphylococcal LOS, and infants with *Escherichia coli* LOS. B) Sample-level RA (%) of g_*Escherichia–Shigella*, g_*Klebsiella*, and g_*Staphylococcus* spp. across four time intervals preceding late-onset sepsis or the corresponding t = 0 of control infants. Boxplots show median and interquartile range (Q1–Q3); points indicate individual samples. Panels depict Controls, all LOS, *S. aureus*-LOS, non-staphylococcal LOS, and *E. coli*-LOS.

In the DC, the preterm microbiota of control infants showed only minor temporal changes (Figure 5A). In contrast, LOS-affected infants showed a more dynamic microbiota composition, characterized by *Escherichia/Shigella* (RA rising from 26% to 41%) and a sharp decline in *Staphylococcus* spp. (RA from 31% to 1%) towards LOS onset. These changes were also seen in the subgroups (e.g., *E. coli-*LOS), where *Escherichia/Shigella* makes up 89% of the gut microbiota in the time interval closest to the onset of LOS (Figure 5A). *Klebsiella* was present across all subgroups, but was almost absent in *E. coli*-LOS. In *S. aureus*-LOS, *Klebsiella* and *Yersinia* increased while *Staphylococcus* spp. decreased, and *Escherichia/Shigella* was near absent (Figure 5B). LMM analyses demonstrated that *Escherichia/Shigella* RA was significantly increased in non-staphylococcal LOS and *E. coli*-LOS (overall group effect), with significant between-group differences at all time periods up to 10 days before onset (FDR-corrected *P* < 0.05) (Table S9). Given the *Escherichia/Shigella* enrichment prior to *E. coli*-LOS, we additionally visualized the non-staphylococcal LOS subgroup excluding the infants with *E. coli*-LOS. After removal of the *E. coli*-LOS cases, *Escherichia/Shigella* remained the predominant genus (Figure S5).

### Differentiating fecal microbiota taxa between preterm infants with LOS and controls in the Discovery Cohort

To identify microbial features differentiating LOS subgroups from controls, we performed MaAsLin2 on microbial ASV data, accounting for multiple and varying number of fecal samples per infant. Significant associations are summarized in Table S10 with the arrows indicating the direction of the association. A varying number of ASVs were linked to LOS status across subgroups. *Enterobacteriaceae* (↑)*, Escherichia/Shigella* (↑), Yersiniaceae (↕), and *Enterobacterales* (↑) were most strongly associated with the overall LOS group. Similar associations were observed in the non-staphylococcal and *E. coli*-LOS subgroups, with 51 *Escherichia/Shigella* ASVs in infants with *E. coli*-LOS. In contrast, in infants with *S. aureus*-LOS, key differentiating ASVs were annotated to *Yersinia* (↑), *Yersiniaceae* (↑), *Ralstonia* (↓), and *Serratia* (↕). Overall, these findings demonstrate distinct microbiota taxa associated with the different subgroups.

### Reproducibility of findings in validation cohorts

We evaluated the reproducibility of the findings from the DC in VC1 and VC2. The *α*-diversity findings were not reproduced in VC1 (Figure S6) and VC2, except for significant differences between *S. aureus*-LOS and controls in VC2 (Figure S7). However, when LMM was applied, the reduced *α*-diversity in *S. aureus*-LOS was no longer apparent, with the exception of the time period −4 to −6 days for Observed and Chao1 (*P* = 0.025 and *P* = 0.030, respectively; Table S9). When investigating the effect of other clinical factors on *α*-diversity, the presence of an invasive device before LOS was significantly associated with both Observed and Shannon in VC1, the mode of delivery with Observed in VC1, and the sample week of life with Shannon in VC1 (Figures S8 and S9).

*β*-diversity findings were consistent across both validation cohorts (Figures S10 and S11), showing similar subgroup clustering with microbiota variation primarily explained by blood culture-isolated pathogen in the pooled analysis (R^2^ = 16.3% and 12.7% in VC1/2, respectively), and time-binned analysis (R^2^ = 17% to 42% and 17% to 30% in VC1/2, respectively) (Figure S3, S12, and S13). Furthermore, in VC1, all clinical parameters, including LOS-associated parameters, contributed to variability, whereas no significant associations besides blood culture-isolated pathogen were observed in VC2 (Figure S4).

The temporal increase in the *Escherichia/Shigella* RA observed in all combined LOS-affected infants in the DC was replicated in VC1, but not in VC2. Instead, *Klebsiella* increased towards LOS onset (Figure S14A). Similar to the DC, *Escherichia/Shigella* dominated the microbiota of infants with *E. coli*-LOS in VC1 and VC2 (Figure S14A), and the presence of *Staphylococcus* spp. and *Klebsiella* was limited in VC2 (Figure S14B). The absence of *Escherichia/Shigella* was replicated in VC1 in the infants with *S. aureus*-LOS, but not in VC2 (Figure S14B). The increase in *Klebsiella* and *Yersinia* and decrease in *Staphylococcus* spp. in *S. aureus*-LOS observed in the DC were not replicated in VC1 and VC2. LMM results were partly replicated. Although *Escherichia/Shigella* showed a significant overall group effect for *E.coli-*LOS in both VC1 and VC2, and for non-staphylococcal LOS in VC1 (FDR-corrected *P* < 0.05), this effect was not consistently observed across all individual time periods (Table S9).

None of the MaAsLin2 associations were reproduced in the validation cohorts (Table S10).

### Late-onset sepsis risk stratification based on core microbiota features

Lastly, we developed two separate RF models for early risk stratification to distinguish non-staphylococcal LOS (model 1) and *E. coli*-LOS (model 2) from controls using the core species-level microbiota features. These subgroups were chosen as they exhibited the most distinct microbiota signatures compared to controls. Both models were trained in the DC and evaluated in VC1 and VC2.

**Figure 6.**
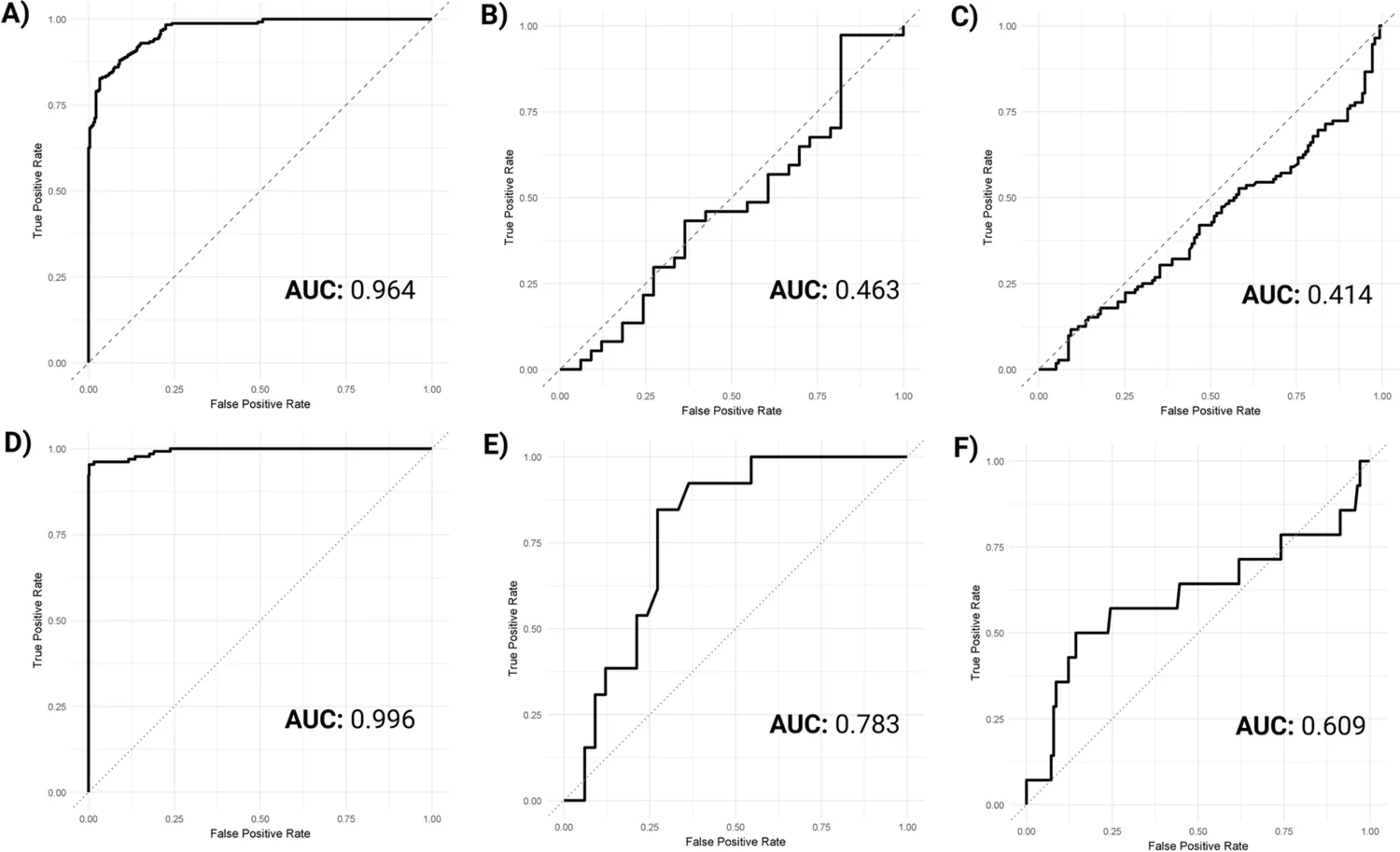
Receiver operating characteristics curve illustrating the performance of the non-staphylococcal and *E. coli* late-onset sepsis (LOS) prediction model. The ROC curve plots sensitivity (true positive rate) against 1 − specificity (false positive rate) across varying classification thresholds. The area under the curve (AUC) quantifies the overall discriminatory ability of the model, with higher AUC values indicating better performance. Panel 6 A, 6B, and 6 C display the ROC curve of the non-staphylococcal LOS model in the Discovery Cohort, Validation Cohort 1, and Validation Cohort 2, respectively. Panels 6D, 6E, and 6F display the ROC curves of the *E. coli-LOS* model in the Discovery Cohort, Validation Cohort 1, and Validation Cohort 2, respectively.

The non-staphylococcal LOS model achieved high performance in the DC (AUROC 0.96, specificity 88% ± 10%, and sensitivity 87% ± 11%) (Table 4, Figure 6). Top contributing features included Gram-positive cocci (e.g., *Staphylococcaceae* and *Streptococcaceae* families) and gut-associated anaerobes (e.g., *Bacteroides* and *Veillonella* species) (Table S11). The model showed poor generalizability in VC1 and VC2 (AUROC 0.46 and 0.41, respectively; Table 4, Figure 6). Additionally, PR curve analysis demonstrated a PR-AUC of 0.97 in the DC (baseline 0.47), compared to 0.50 (baseline 0.53) and 0.42 (baseline 0.45) in VC1 and VC2, respectively (Figure S15).

**Table 4.** Early late-onset sepsis risk stratification based on core microbiota features to discriminate non-staphylococcal late-onset sepsis (LOS) from controls. This table displays the results of random forest model used to differentiate discriminate non-staphylococcal LOS from controls in the three cohorts. The core microbiota, defined as ASVs from the overlapping bacterial species between the three cohorts, were used as input for the model. The model was developed in DC using a ten-fold cross-validation. The results displayed represent the average results across all folds. Next, the trained model was evaluated in VC1 and VC2, with the corresponding results presented in this table.

	Precision	F1 score	Specificity	Sensitivity	AUROC
Discovery Cohort					
*Average*	0.880	0.867	0.884	0.868	0.964
*SD*	0.093	0.064	0.099	0.107	0.029
Validation Cohort 1					
*Average*	0.473	0.487	0.393	0.486	0.463
Validation Cohort 2					
*Average*	0.396	0.508	0.136	0.705	0.414

To assess whether a pathogen-specific model could improve performance, we developed a second model specifically for *E. coli-*LOS, which demonstrated high accuracy in the DC (AUROC 1.00 ± 0.00, specificity 100% ± 0%, and sensitivity 88% ± 11%) (Table 5, Figure 6), with key features from the Enterobacterales order (*Enterobacteriaceae*, *Yersiniaceae*), and *Escherichia/Shigella* (Table S12). In VC1 and VC2, AUROC of 0.78 and 0.61 were achieved, respectively (Table 5, Figure 6). PR curve analysis for the *E. coli*-LOS model revealed a PR-AUC of 0.98 in DC (baseline 0.18), 0.46 (baseline 0.28) in VC1, and 0.22 (baseline 0.09) in VC2 (Figure S16).

**Table 5.** Early late-onset sepsis risk stratification based on core microbiota features to discriminate potential *Escherichia coli* late-onset sepsis (LOS) from controls. This table displays the results of the random forest model used to differentiate *E. coli*-LOS from controls in the three cohorts. The core microbiota, defined as ASVs from the overlapping bacterial species between the three cohorts, were used as input for the model. The model was developed in DC using a three-fold cross-validation. The results displayed represent the average results across all folds. Next, the trained model was evaluated in VC1 and VC2, with the corresponding results presented in this table.

	Precision	F1 Score	Specificity	Sensitivity	AUROC
Discovery Cohort					
*Average*	1	0.935	1	0.884	0.996
*SD*	0	0.063	0	0.108	0.007
Validation Cohort 1					
*Average*	nan	nan	0	1	0.783
Validation Cohort 2					
*Average*	0.25	0.267	0.914	0.286	0.609

Relative to their respective baselines, the *E. coli*-LOS model consistently exceeded the no-skill threshold across all cohorts (VC1: +0.18, VC2: +0.13; Figure S16), whereas the non-staphylococcal LOS model performed at or below baseline in both validation cohorts (VC1: −0.03, VC2: −0.03; Figure S15), indicating marginally better relative PR-AUC performance for the *E. coli*-LOS model.

## Discussion

This study aimed to identify LOS-specific microbiota features as well as develop and validate an early microbiota-based LOS risk-stratification model. We detected significant microbiota shifts up to 10 days before LOS onset, which were most pronounced in infants with *E. coli*-LOS. Opposed to the multi-pathogen non-staphylococcal LOS model, the pathogen-specific *E. coli*-LOS model showed a modest improvement in predictive ability across cohorts, suggesting that stratification by causative pathogen may be a promising avenue for clinically meaningful microbiota-based risk stratification strategies. Yet, the limited predictive performance across validation cohorts also reveals the challenges of developing microbiota-based prediction tools that are robust and generalizable across cohorts.

A dynamic microbiota composition preceding non-staphylococcal LOS was observed in the DC, characterized by an increase in *Escherichia/Shigella*. However, this pattern was not observed before *S. aureus*-LOS onset; the preceding microbiota consisted of minimal *Escherichia/Shigella* and no rise in *Staphylococcus* spp. Similar trends were observed in VC1/VC2, although these effects were less pronounced. These findings suggest that non-staphylococcal LOS may be more strongly associated with gut microbiota perturbations, aligning with studies reporting higher discriminatory accuracy for gram-negative LOS prediction compared to *S. aureus* and CoNS-LOS using fecal volatile organic compounds.^32^ Although the non-staphylococcal LOS group in the three cohorts also included Gram-positive pathogens (e.g., *Enterococcus faecalis*, Group B *Streptococcus*), the majority of cases were caused by Gram-negative pathogens, supporting the alignment of our results with previous research.

In line with previous studies,^33‐35^ infants with LOS exhibited consistently reduced *α*-diversity across all subgroups in the DC. However, this longitudinal pattern was not replicated in the validation cohorts. This variability across cohorts and studies suggests that *α*-diversity alone is unlikely to represent a robust or generalizable biomarker for LOS. The increase in *Escherichia/Shigella* demonstrated in the DC may result from reduced *α*-diversity preceding LOS, facilitating overgrowth of opportunistic bacteria such as *E. coli*. Such overgrowth, potentially driven by virulence-associated inflammation, may promote translocation across the immature gut barrier into the bloodstream.^36‐38^ These findings support gut-derived LOS, but the absence of blood isolate sequencing precludes strain-level analysis, prohibiting definitive conclusions regarding causality. *β*-diversity analysis revealed that patient ID explained most microbiota variation across all cohorts (R^2^ = 53% in DC), indicating strong interindividual variety, an earlier documented phenomenon in preterm infants.^39^ Stratification based on blood culture-isolated pathogen explained the next greatest variation across all cohorts (Pooled: R^2^ = 12.7%, time-binned: R^2^ = 14% to 30% in DC) among all analyzed clinical parameters. Despite pronounced interindividual variability, strong pathogen-specific microbiota signatures were observed that remained detectable, even longitudinally, and were replicated across validation cohorts.

We used ML to explore the potential of a microbiota-based LOS prediction model. Both RF models demonstrated excellent discrimination in the DC (non-staphylococcal LOS: AUROC 0.96; *E. coli*-LOS AUROC 1.00). However, these scores likely reflect overfitting given the small sample size. For the non-staphylococcal LOS model, the predictive accuracy decreased markedly in both validation cohorts, demonstrating poor generalizability. PR-AUC analyses, which are better suited for imbalanced datasets, corroborated this pattern, with performance at or below the no-skill baseline, indicating no meaningful predictive utility of the non-staphylococcal LOS model. In contrast, the *E. coli*-LOS model consistently exceeded the no-skill baseline by 0.18 and 0.13 in VC1 and VC2, respectively, indicating that pathogen-specific modeling may retain a modest but consistent predictive signal across cohorts. Increased abundance of *Escherichia/Shigella* in *E. coli*-LOS-affected infants represents a consistent, biologically meaningful microbial signature that replicated across cohorts and different analytical approaches, supporting LOS stratification by causative pathogen. Nonetheless, this signal did not readily translate into a fully generalizable predictive model, highlighting the limitations of microbiota-based ML approaches.

Several factors may explain the limited generalization from the DC to the validation cohorts. At the sample level, the small number of cases within the subgroups may have restricted stable model training and increased the risk of overfitting. Differences in pathogen distribution may further result in limited validation performance for the non-staphylococcal model. At the technical level, variation in DNA extraction (e.g., with/without bead-beating procedures) and primer selection can introduce variability, complicating cross-study comparison.^40^ ^,^ ^41^ Although bioinformatics workflows were harmonized,^41‐43^ these methodological differences warrant cautious interpretation of findings. Additionally, differences in storage time between cohorts and storage temperature at −20 °C may have affected DNA quality and microbiota composition.^44^ ^,^ ^45^ Lastly, a matched case‒control design was used to mitigate the impact of several microbiota-modulating factors. Direct adjustment of the RF models for additional potential confounders, for example, mode of delivery or antibiotic administration, may possibly improve generalization. As associations between potential confounders and microbiota composition differed across cohorts, no single confounder could be uniformly selected for adjustment without risking introducing additional heterogeneity between cohorts.

Strengths of this study include its multicenter design with a relatively large sample size, prospective sampling design, detailed data collection, and longitudinal preclinical microbiota analysis. Subgroup analysis allowed for discrimination of *E. coli*-specific LOS patterns and the inclusion of two validation cohorts with different methodological approaches aided in the distinction of true microbial LOS signatures instead of cohort-specific findings. Although this may have introduced variability, consistent microbiota-associated LOS signals remained detectable. Other limitations include the small sample size for individual pathogens, with only *E. coli* represented across all three cohorts. This precludes the development of predictive pathogen-specific models for other pathogens (e.g., *Klebsiella* spp., *Serratia marcescens)* despite similar pre-LOS onset patterns observed previously.^7^ The absence of strain-level analysis of the gut microbiota and the blood-cultured pathogen restricts our observations to microbial compositional changes rather than pathogen origin. Additionally, species-level interpretation of 16S rRNA amplicon data is limited, particularly for closely related taxa such as *Staphylococcus* spp., and when using short amplicons such as the V4 region.^46^ ^,^ ^47^ Species-level assignments should therefore be interpreted with caution, and future studies using species-resolving methods are needed to confirm signals at the species level.

The cohort-specific differences underscore the complexity of translating microbiota-based risk models across cohorts and highlight the need for rigorous validation before clinical application. Future studies should increase sample size (e.g., broadening inclusion criteria with careful confounder control, increasing case/control ratio, and international collaborations) to enhance diversity and size of underrepresented LOS subgroups. Although we identified a LOS-associated microbial signal despite substantial methodological heterogeneity across cohorts, future microbiota studies should prospectively harmonize DNA extraction protocols, sequencing strategy, and bioinformatic processing, in addition to sample collection and storage conditions, before patient recruitment. Such standardization is essential to reduce technical variation, improve comparability across centers and cohorts, and enable more robust microbiota-based risk stratification. Future models may also benefit from real-time integration of clinical data (e.g., continuous vital parameters). Additionally, future work should extend beyond taxonomy by incorporating metagenomics to characterize the resistome and virulence factors, or metabolomic analyses to assess functional microbial output. Specially for *E. coli-*LOS, which is associated with substantial morbidity and mortality,^48^ ^,^ ^49^ the potential of microbiota-based early risk stratification is encouraging. Implementation of an *E. coli*-LOS risk model could reduce disease burden by enabling timely and targeted interventions while limiting the reliance on untargeted broad-spectrum antibiotics, in case of clinical suspected LOS.^8^ Integration of rapid microbial profiling techniques into clinical workflows (even at the bedside) may further improve feasibility, as current sequencing methods are limited by long turnaround times and high costs.^50^

In conclusion, stratifying LOS by causative pathogen rather than pooling all LOS cases into a single heterogeneous group represents a more informative framework for microbiota-based risk prediction. This approach yielded the strongest discriminatory ability for *E. coli*-LOS compared to the multi-pathogen non-staphylococcal LOS model. Substantial variability across cohorts remained, and generalization of the *E. coli*-LOS model from the DC to validation cohorts was limited. While our findings suggest that pathogen-specific microbiota patterns, particularly for *E. coli*-LOS, may hold promise as a noninvasive biomarker for microbiota-based LOS risk stratification, further validation in larger and methodologically harmonized cohorts is required. Only with such evidence can the potential clinical utility of early microbiota-based risk assessment, and its possible role in supporting timely and targeted interventions, be appropriately evaluated.

## Supplementary Material

Supplementary Material Supplementary material.docx

## Data Availability

Data supporting this study are deposited in Figshare. Sequencing data can be found at: https://doi.org/10.6084/m9.figshare.31293145. The analysis scripts are available through: https://doi.org/10.6084/m9.figshare.31293511.
