# Supplementary material for: Early risk stratification of late-onset sepsis in very preterm infants by intestinal microbiota profiling: a multicenter case–control validation study
Source: Gut Microbes. 2026 Jul 2;18(1):2693365. doi: 10.1080/19490976.2026.2693365 (PMC13336302; doi:10.1080/19490976.2026.2693365)
Supplement: Supplementary Material — docx [file KGMI_A_2693365_SM1316.docx]

**Supplementary materials**

# Corresponding manuscript: *Early risk stratification of late-onset sepsis in very preterm infants by intestinal microbiota profiling: a multicenter case-control validation study*

Authors: Nina M. Frerichs*, Rimke R. de Kroon*, Yannick van Schajik, Sofia el Manouni el Hassani, Aranka J. van Wesemael, Willem P. de Boode, Veerle Cossey, Christian V. Hulzebos, Chris H.P. van den Akker, Marlou M.A. Raets, Esther d’Haens, Daniel C. Vijlbrief, Mirjam M. van Weissenbruch, Wouter J. de Jonge, Nanne K. de Boer, Johannes B. van Goudoever, Andrew Beggs, Mohammed Nabil Quraishi, Mark Davids, Sudip Mondal, Animesh Acharjee, Hendrik J. Niemarkt, Tim de Meij

*Contributed equally to the project

**APPENDIX A**

Below a detailed explanation is provided for the matching criteria, sample preparation, DNA extraction, gene amplification, and library preparation & sequencing for each cohort (DC, VC1, and VC2, respectively).

***Discovery Cohort (DC)***

*Sample selection*

In 2019, infants with LOS were included from participating NICUs between January 2017 and November 2018 (Figure 2). Control infants were matched with LOS cases based on center of birth, GA (± 5 days), BW (± 150 grams), and postnatal age at LOS onset (± 0 days). Fecal samples collected up to 10 days prior to clinical onset of LOS (t-1 to t-10) were selected for microbiota analysis. Infants were included in the study if at least 2 samples within this time period were available for analysis.

*Sample preparation*

The fecal were shipped inside their original collection tube on dry ice from Amsterdam UMC, location VUMC (Amsterdam, The Netherlands) to the Institute of Cancer and Genomic Sciences at The University of Birmingham (Birmingham, United Kingdom) and stored at -80°C. Prior to DNA extraction, the samples were thawed on ice and weighted on a calibrated scale (AL204 Analytical Balance, Mettler Toledo, OH, USA). A maximum of 250 mg was used for analyses.

*DNA extraction*

The DNA was isolated using the QIAamp PowerFecal DNA Kit (Qiagen, Hilden, Germany) with minor adjustments to the protocol. For the sample disruption step, the TissueLyser II (Qiagen, Hilden, Germany) was used for 2 minutes at 30 Hz. To elute the DNA, 50 μl of elution buffer was used. Negative control samples were included in the DNA extraction steps. The DNA was stored at 2-8°C prior to quantification and PCR. DNA quantification was done using the Invitrogen QubitTM 3 Fluorometer with the double strand DNA (dsDNA) BR (Broad Range) Assay Kit (Thermo Fisher Scientific, Waltham, MA, USA). DNA was normalized to 5 ng/μl prior to performing PCR.

*Gene amplification*

16S rRNA gene amplification and sequencing was done using the Earth Microbiome Project Protocol by the department of Cancer and Genomic Sciences at the University of Birmingham (20, 21). The V4 region of the 16S rRNA gene was amplified with a custom made 515F forward primer (Sigma-Aldrich, Saint Louis, MA, USA) and 806R reverse primer (Sigma-Aldrich, Saint Louis, MA, USA) by using a one-step, single-indexed PCR approach. A total volume of 50 μl for each PCR reaction was used consisting of 47 μl Mastermix (34.6 μl nuclease free H2O (Qiagen, Hilden, Germany), 10 μl 5x Platinum II PCR Buffer (Thermo Fisher Scientific, Waltham, MA, USA), 1 μl dNTP Mix (Thermo Fisher Scientific, Waltham, MA, USA), 1 μl Reverse Primer (10 μM final concentration), 0.4 μl PlatinumTM II *Taq* Hot-Start DNA Polymerase (Thermo Fisher Scientific, Waltham, MA, USA), 2 μl 5ng/μl DNA and 1 μl Forward Primer (10 μM final concentration). PCR settings were as follows; 94°C for 3 min, 35 cycles: 94°C for 45s/50°C for 60s/72°C for 90s, 72°C for 10 min and hold at 4°C. The DNA extraction and PCR reactions were both performed with appropriate controls. The cleanup was performed using AMPure XP magnetic beads (A63882, Beckman Coulter, Brea, CA, USA) as per protocol with minor adjustments; the washing steps were performed with fresh 80% ethanol and for DNA elution the elution buffer from the QIAamp PowerFecal DNA Kit (Qiagen, Hilden, Germany) was used (22).

*Library preparation & sequencing*

The samples were quantified with the Invitrogen Qubit^TM^ 3 Fluorometer using the dsDNA HS (High Sensitivity) Assay Kit (Thermo Fisher Scientific, Waltham, MA, USA), normalized and pooled to create a library. Subsequently, the library was paired-end sequenced (2x250bp) on an Illumina MiSeq platform (Illumina, San Diego, CA, USA).

***Validation Cohort 1 (VC1)***

VC1 consist of samples previously analyzed by a novel molecular bacterial detection technique (IS-pro, InBiome, the Netherlands), as described previously (15). In short, preterm infants with LOS were included from participating NICUs between May 2014 and December 2016 (Figure 2). Control infants were matched to LOS infants based on center of birth and postnatal age at LOS onset. Fecal samples collected up to 5 days prior to clinical onset of LOS (t-1 to t-5) were selected for microbiota analysis. Infants were included in the study if at least 2 samples within this time period were available for analysis (15). The isolated DNA was shipped on dry ice to the Institute of Cancer and Genomic Sciences at The University of Birmingham. DNA quantification, gene amplification, library preparation & sequencing was conducted, following the same protocol as described for the DC.

***Validation Cohort 1 (VC2)***

*Sample selection*

Infants with LOS and controls were include from participating NICUs between April 2016 and January 2017 (Figure 2). Control infants were matched with LOS cases based on center of birth, GA (± 5 days), BW (± 150 grams), and postnatal age at LOS onset (± 0 days). Fecal samples collected up to 10 days prior to clinical onset of LOS (t-1 to t-10) were selected for microbiota analysis. Infants were included in the study if at least 2 samples within this time period were available for analysis.

*Sample preparation*

The fecal samples were shipped inside their original collection tube on dry ice for Amsterdam UMC, location VUMC (Amsterdam, The Netherlands) to Tytgat Institute for Liver and Intestinal research (Amsterdam, The Netherlands) and stored at -20°C until DNA extraction. Prior to DNA extraction, the samples were thawed on ice and ~250 mg feces was used for further analysis.

*DNA extraction*

The DNA was isolated using the PSP Spin Stool DNA Plus Kit (Invitek Molecular, Berlin, Germany) with minor adjustments to the protocol. The fecal samples were homogenized in Stool DNA Stabilizer buffer (Isogen Life Sciences, Utrecht, the Netherlands) by using glass and ceramic based lysing beads (Lysing Matrix E 2mL tubes, MP Biomedicals^TM^, Santa Ana, CA, USA) and Precellys 24 Touch Homogenizer (3x 30s, 6.5 ms-2, Bertin Technologies, Montigny-le-Bretonneux, France). Subsequently, the suspension was heated (15 minutes at 95°C). The supernatant was transferred to the PSP InviAdsorb tubes, as provided by the manufacturer, and manufacturer’s kit protocol was followed from this step onwards. Negative control samples were included in the DNA extraction steps. The DNA was eluted in 50 µl DNAse free water. DNA quantification was done using Nanodrop ND-1000 (Thermo Fisher Scientific, Waltham, MA, USA).

*Gene amplification*

16S rRNA gene amplification and sequencing was performed by the Microbiota Centre Amsterdam (MiCA, Amsterdam, the Netherlands), as previously described (23). An adapted one-step PCR method was used to amplify the barcoded V3–V4 region of the 16S rRNA gene (341 forward and 805 reverse primer) (24, 25). Twenty ng of DNA was used in the PCR mix; 6μL 5× HF buffer (Thermo Fisher Scientific, Waltham, MA, USA), 0.75μL PCR Grade Nucleotide Mix (10 μM) (Thermo Fisher Scientific, Waltham, MA, USA), 0.3μL Phusion DNA Polymerase (2 U/µL), 18.95μL nuclease free water, 1.5μl forward Index Primer (10 μM) and 1.5μl reversed Index Primer (10μM). The amplification program was as follows: initial denaturation at 98°C for 30s; 25 cycles of denaturation at 98°C for 10s, annealing at 55°C for 20s, elongation at 72°C for 90s; and an extension at 72°C for 10 min (24). PCR was performed using a Biometra Thermocycler (Göttingen, Germany). To confirm the presence of the PCR product 1% agarose gel electrophoresis containing ethidium bromide was used. The Biomek FX robot with AMPure XP beads (1.8x) were used for clean-up of the amplified product according to manufacturer’s guidelines (Beckman Coulter, Brea, CA, USA).

*Library preparation & sequencing*

DNA concentrations were quantified using the Qubit^TM^ dsDNA BR Assay Kit (Thermo Fisher Scientific, Waltham, MA, USA). The quality of the pooled library was assessed using High Sensitive DNA chip on the Bioanalyzer 21000 (Agilent, Santa Clara, CA, USA). The purified samples, each labeled with a unique index, were equimolarly pooled and sequenced on the Illumina MiSeq platform (Illumina, San Diego, CA, USA) using the MiSeq V3 – 600 cycle kit. Paired-end sequencing (2x250 bp) was performed to generate the sequencing data (24).

**SUPPLEMENTARY TABLES**

**Table S1. Clinical data extracted from electronic patient files**

| **Variable** | **Definition / Details** |
| --- | --- |
| Biological sex | Female or male |
| Mode of delivery | Vaginal or cesarean section |
| Gestational age | Weeks + days |
| Birth weight | Grams |
| Multiple gestation | Singleton or multiple |
| Apgar score | Score (1-10) at 5 minutes postpartum |
| Duration of NICU stay | Days |
| 29-day mortality | Death within first 29 days of life |
| Antenatal corticosteroids | None, incomplete course, or complete course |
| Surfactant administration | Single or multiple doses given within first 72h of life, |
| Antibiotic use | Ratio of days with antibiotic administration during first 29 days of life, as well as ratio of antibiotic administration prior to t=0 |
| Postnatal age at full enteral feeding | Day when parenteral nutrition and intravenous glucose were ceased |
| Feeding type | Type on the day of full enteral feeding, categorized as: exclusive human milk (mothers own milk or donor human milk), exclusive formula or mixed (human milk + formula), regardless of additives. |
| Exposure to formula | Binary variable: yes/no during first 29 days of life. |
| Day of life of blood-culture confirmed sepsis (t=0) | Day of which diagnostic work-up is performed that resulted in sepsis diagnosis, including at least a blood culture. |
| Invasive medical devices (48 h prior to t*=*0) | Presence of endotracheal tube, central vascular catheter (including umbilical venous/arterial), peripheral catheter or invasive ventilation. |

**Table S2. Median number of 16S reads per week of life per cohort**

|  | **Week 1** | | **Week 2** | | **Week 3** | | **Week 4** | |
| --- | --- | --- | --- | --- | --- | --- | --- | --- |
|  | **16S reads** | **n** | **16S reads** | **n** | **16S reads** | **n** | **16S reads** | **n** |
| **Discovery cohort** | 51761 | 60 | 52705 | 95 | 47906 | 47 | 48322 | 13 |
| **Validation Cohort 1** | 34446 | 22 | 110293 | 31 | 99906 | 6 | 38106 | 11 |
| **Validation Cohort 2** | 14950 | 92 | 16172 | 102 | 15149 | 53 | 9225 | 4 |

**Table S3. Overview of included samples per cohort**

|  | **Time interval** | | | | **Total samples** |
| --- | --- | --- | --- | --- | --- |
|  | 1-3 days | 4-6 days | 7-8 days | 9-10 days |  |
| **Discovery Cohort (DC)** | | | | | |
| *Total number of samples* | 62 | 61 | 50 | 43 | 216 |
| *Control samples* | 41 | 32 | 23 | 19 | 115 |
| *LOS samples* | 21 | 29 | 27 | 24 | 101 |
| *Samples per individual, median (min, max)* | 2 (1, 3) | 2 (1,3) | 1 (1,2) | 1 (1,2) | 5 (1,10) |
| *Mean storage duration (years)* | - | - | - | - | 2.01 |
| **Validation Cohort 1 (VC1)** | | | | | |
| *Total number of samples* | 49 | 21 | n.a. | n.a. | 70 |
| *Control samples* | 23 | 10 | n.a. | n.a. | 33 |
| *LOS samples* | 26 | 11 | n.a. | n.a. | 37 |
| *Samples per individual, median (min, max)* | 2 (1,3) | 1 (1,2) | n.a. | n.a. | 3 (1, 5) |
| *Mean storage duration (years)* | - | - | - | - | 1.17 |
| **Validation Cohort 2 (VC2)** | | | | | |
| *Total number of samples* | 95 | 81 | 46 | 29 | 251 |
| *Control samples* | 54 | 48 | 22 | 15 | 139 |
| *LOS samples* | 41 | 33 | 24 | 14 | 112 |
| *Samples per individual, median (Q1, Q3)* | 2 (2,3) | 2 (2,3) | 2 (1,2) | 2 (1,2) | 6 (4, 8) |
| *Mean storage duration (years)* | - | - | - | - | 7.11 |

**Table S4. Preprocessing procedures and sample dropouts per cohort**

|  | **Discovery Cohort** | **Validation Cohort 1** | **Validation Cohort 2** |
| --- | --- | --- | --- |
| *Total fecal samples available (total ASVs available)* | 216 (4100) | 70 (6120) | 251 (306) |
| *Fecal samples (ASVs) remaining after removal of non-bacterial ASVs* | 216 (4052) | 70 (6077) | 251 (305) |
| *Fecal samples (ASVs) remaining after removal of samples <10.000 total reads* | 210 (4052) | 66 (6077) | 196 (305) |
| *Fecal samples (ASVs) remaining after removal of ASVs with >80% zero values* | 210 (368) | 66 (384) | 196 (25) |
| *Summary -Number of samples from control group (%)*  *-Number of samples from disease group (%)*  *-Median number of samples per infant* | 114 (54.3%)  96 (45.7%)  6 | 31 (46.3%)  36 (53.7%)  3 | 117 (59.7%)  79 (40.3)  3 |

**Table S5. Discovery Cohort demographics**

|  | **Late-onset sepsis (n=18)** | **Controls (n=18)** | **p-value** |
| --- | --- | --- | --- |
| **Baseline characteristics** | | | |
| Gestational age (days), median [Q1-Q3] | 185 [177-196] | 184 [178-200] | 0.692 |
| Birth weight (grams), median [Q1-Q3] | 905 [684-1056] | 916 [748-1094] | 0.496 |
| Biological sex (Female, n [%]) | 5 [28] | 10 [56] | 0.091 |
| Multiple births (Singleton, n [%]) | 12 [67] | 14 [78] | 0.457 |
| Apgar score 5 min, median [Q1-Q3] | 8 [6-8] | 7 [6-8] | 0.987 |
| Mode of delivery (Vaginal n [%]) | 8 [44] | 4 [22] | 0.157 |
| Mortality (Yes, n [%]) | 1 [6] | 0 [0] | 0.310 |
| Length of NICU stay (days), median [Q1-Q3] | 53 [32-86] | 43 [28-67] | 0.367 |
| Day of life blood-culture confirmed sepsis, median [Q1-Q3] | 15 [12-20] | N/A | N/A |
| **Medication practices** | | | |
| Ratio of antibiotic administration^1^ in first 29 days of life, median [Q1-Q3] | 0.62 [0.51-0.66] | 0.26 [0.10-0.37] | <0.001 |
| Ratio of antibiotic administration before t=0, median [Q1-Q3] | 0.25 [0.21-0.33] | 0.33 [0.16-0.59] | 0.590 |
| Exposure to antibiotics prior to t=0^2^ (Yes, n [%]) | 16 [89] | 16 [89] | 1.000 |
| Surfactant administration in the first 4 days of life, n [%] (2 missing) |  | | |
| *None* | 2 [11] | 4 [25] | 0.566 |
| *1x surfactant administration* | 5 [28] | 4 [25] |  |
| *2x or more surfactant administration* | 11 [61] | 8 [50] |  |
| Antenatal corticosteroids, n [%] |  |  | |
| *None* | 6 [33] | 7 [39] | 0.168 |
| *Incomplete course* | 6 [33] | 6 [33] |  |
| *Complete* | 6 [33] | 5 [28] |  |
| **Average feeding practice** | | | |
| Day of life on reaching full enteral feeding^3^, median [Q1-Q3] (4 missing) | 12 [9-18] | 10 [8-14] | 0.203 |
| Feeding type category at day of life on reaching full enteral feeding, n [%] (6 missing) |  |  | |
| *Exclusively human milk^4^* | 10 [63] | 11 [79] | 0.338 |
| *Exclusively formula feeding* | 0 [0] | 0 [0] |  |
| *Mix of human milk and formula feeding* | 6 [37] | 3 [21] |  |
| Received formula feeding in the first 29 days (Yes [%]) (6 missing) | 11 [73] | 10 [67] | 0.690 |
| Reached full enteral feeding at t=0 (Yes, n [%]) (4 missing) | 12 [71] | 13 [87] | 0.272 |
| Parenteral feeding before t=0 (days), median [Q1-Q3] (2 missing) | 11 [9-15] | 9 [8-13] | 0.189 |
| **Exposure to invasive medical devices** | | | |
| Exposure to invasive medical device in 48 hours prior to t=0 (Yes, n[%]) | 15 [83] | 11 [61] | 0.137 |
| Peripheral IV | 13 [72] | 10 [56] | 0.298 |
| Central catheter | 6 [33] | 4 [22] | 0.457 |
| Invasive ventilation | 5 [28] | 4 [22] | 0.700 |
| Invasive ventilation before t=0 (days), median [Q1-Q3] | 5 [0-15] | 3 [0-9] | 0.196 |
| ^1^The ratio of antibiotic administration was calculated as the number of days with documented antibiotic use divided by 29, representing the proportion of the first 29 days exposed to antibiotics. ^2^The day on which the diagnostic work-up resulting in the LOS diagnosis was performed (and the corresponding matched day for the control infant) was defined as t=0. ^3^Full enteral feeding was defined as the first day on which both parenteral nutrition and intravenous glucose were ceased. ^4^Human milk was defined as mother’s own milk, donor human milk, or a combination. *Abbreviations: NICU = neonatal intensive care unit.* | | | |

**Table S6. Validation Cohort 1 demographics**

|  | **Late-onset sepsis (n=12)** | **Controls (n=12)** | **p-value** |
| --- | --- | --- | --- |
| **Baseline demographic characteristics** | | | |
| Gestational age (days), median [Q1-Q3] | 187 [183-198] | 194 [191-199] | 0.125* |
| Birth weight (grams), median [Q1-Q3] | 819 [755-1110] | 1058 [921-1174] | 0.175* |
| Biological sex (Female, n [%]) | 6 [50] | 6 [50] | 1.000^#^ |
| Multiple births (Singleton, n [%]) | 8 [67] | 9 [75] | 1.000^^^ |
| Apgar score 5 min, median [Q1-Q3] | 7 [6-8] | 8 [6-9] | 0.573* |
| Mode of delivery (Vaginal n [%]) | 7 [58] | 5 [42] | 0.414^#^ |
| Mortality (Yes, n [%]) | 2 [17] | 0 [0] | 0.478^^^ |
| Length of NICU stay (days), median [Q1-Q3] | 41 [18-61] | 27 [22-40] | 0.435* |
| Day of life blood-culture confirmed sepsis, median [Q1-Q3] | 13 [6-17] | N/A | N/A |
| **Medication practices** | | | |
| Ratio of antibiotic administration^1^ in first 29 days of life, median [Q1-Q3] | 0.58 [0.32-0.73] | 0.16 [0.08-0.23] | <0.001* |
| Ratio of antibiotic administration before t=0, median [Q1-Q3] | 0.23 [0-0.46] | 0.27 [0.10-0.50] | 0.641* |
| Exposure to antibiotics prior to t=0^2^ (Yes, n [%]) | 8 [67] | 10 [83] | 0.640^^^ |
| Surfactant administration in the first 4 days of life, n [%] (2 missing) |  | | |
| *None* | 1 [9] | 0 [0] | 0.543^#^ |
| *1x surfactant administration* | 3 [27] | 2 [20] |  |
| *2x or more surfactant administration* | 7 [64] | 8 [80] |  |
| Antenatal corticosteroids, n [%] |  | | |
| *None* | 3 [27] | 8 [67] | 0.124^#^ |
| *Incomplete course* | 4 [36] | 3 [25] |  |
| *Complete* | 4 [36] | 1 [8] |  |
| **Average feeding practice** | | | |
| Day of life on reaching full enteral feeding^3^, median [Q1-Q3] (4 missing) | 11 [10-13] | 8 [8-9] | 0.003* |
| Feeding type category at day of life on reaching full enteral feeding, n [%] (6 missing) |  | | |
| *Exclusively human milk^4^* | 8 [80] | 5 [46] | 0.098^#^ |
| *Exclusively formula feeding* | 0 [0] | 4 [36] |  |
| *Mix of human milk and formula feeding* | 2 [20] | 2 [18] |  |
| Received formula feeding in the first 29 days (Yes [%]) (6 missing) | 9 [82] | 10 [91] | 1.000^^^ |
| Reached full enteral feeding at t=0 (Yes, n [%]) (4 missing) | 5 [45] | 6 [55] | 0.670^#^ |
| Parenteral feeding before t=0 (days), median [Q1-Q3] (2 missing) | 11 [6-12] | 8 [5-9] | 0.316* |
| **Exposure to invasive medical devices** | | | |
| Exposure to invasive medical device in 48 hours prior to t=0 (Yes, n[%]) | 10 [83] | 6 [50] | 0.193^^^ |
| Peripheral IV | 8 [67] | 4 [33] | 0.220^^^ |
| Central catheter | 3 [25] | 3 [25] | 1.000^^^ |
| Invasive ventilation | 1 [8] | 0 [0] | 1.000^^^ |
| Invasive ventilation before t=0 (days), median [Q1-Q3] | 1 [0-2] | 0 [0-1] | 0.364* |
| ^1^The ratio of antibiotic administration was calculated as the number of days with documented antibiotic use divided by 29, representing the proportion of the first 29 days exposed to antibiotics. ^2^The day on which the diagnostic work-up resulting in the LOS diagnosis was performed (and the corresponding matched day for the control infant) was defined as t=0. ^3^Full enteral feeding was defined as the first day on which both parenteral nutrition and intravenous glucose were ceased. ^4^Human milk was defined as mother’s own milk, donor human milk, or a combination. *Abbreviations: NICU = neonatal intensive care unit.* | | | |

**Table S7. Validation Cohort 2 demographics**

|  | **Late-onset sepsis (n=28)** | **Controls (n=28)** | **p-value** |
| --- | --- | --- | --- |
| **Baseline demographic characteristics** | | | |
| Gestational age (days), median [Q1-Q3] | 185 [177-201] | 186 [176-200] | 0.928 |
| Birth weight (grams), median [Q1-Q3] | 883 [700-1015] | 830 [755-1046] | 0.793 |
| Biological sex (Female, n [%]) | 15 [54] | 15 [54] | 1.000 |
| Multiple births (Singleton, n [%]) | 14 [50] | 17 [61] | 0.420 |
| Apgar score 5 min, median [Q1-Q3] | 7 [6-8] | 8 [7-9] | 0.072 |
| Mode of delivery (Vaginal n [%]) | 12 [43] | 12 [43] | 1.000 |
| Mortality (Yes, n [%]) | 1 [4] | 3 [11] | 0.611 |
| Length of NICU stay (days), median [Q1-Q3] | 48 [29-69] | 44 [28-63] | 0.974 |
| Day of life blood-culture confirmed sepsis, median [Q1-Q3] | 12 [9-17] | N/A | N/A |
| **Medication practices** | | | |
| Ratio of antibiotic administration in first 29 days of life, median [Q1-Q3] | 0.69 [0.55-0.79] | 0.26 [0.16-0.41] | <0.001 |
| Ratio of antibiotic administration before t=0, median [Q1-Q3] | 0.39 [0.23-0.62] | 0.43 [0.32-0.57] | 0.850 |
| Exposure to antibiotics prior to t=0 (Yes, n [%]) | 25 [89] | 27 [96] | 0.611 |
| Surfactant administration in the first 4 days of life, n [%] (3 missing) |  | | |
| *None* | 3 [11] | 2 [8] | 0.747 |
| *1x surfactant administration* | 6 [22] | 8 [31] |  |
| *2x or more surfactant administration* | 18 [67] | 16 [62] |  |
| Antenatal corticosteroids, n [%] |  |  | |
| *None* | 7 [25] | 12 [43] | 0.307 |
| *Incomplete course* | 11 [39] | 10 [36] |  |
| *Complete* | 10 [36] | 6 [21] |  |
| **Average feeding practice** | | | |
| Day of life on reaching full enteral feeding, median [Q1-Q3] (6 missing) | 11 [9-15] | 10 [9-12] | 0.171 |
| Feeding type category at day of life on reaching full enteral feeding, n [%] (10 missing) |  | | |
| *Exclusively human milk* | 14 [64] | 19 [79] | 0.410 |
| *Exclusively formula feeding* | 3 [14] | 1 [4] |  |
| *Mix of human milk and formula feeding* | 5 [23] | 4 [17] |  |
| Received formula feeding in the first 29 days (Yes [%]) (4 missing) | 22 [88] | 21 [78] | 0.469 |
| Reached full enteral feeding at t=0 (Yes, n [%]) | 14 [58] | 19 [73] | 0.272 |
| Parenteral feeding before t=0 (days), median [Q1-Q3] | 12 [10-16] | 10 [9-14] | 0.579 |
| **Exposure to invasive medical devices** | | | |
| Exposure to invasive medical device in 48 hours prior to t=0 (Yes, n[%]) | 26 [93] | 22 [79] | 0.252 |
| Peripheral IV | 21 [75] | 15 [54] | 0.094 |
| Central catheter | 10 [36] | 10 [36] | 1.000 |
| Invasive ventilation | 13 [46] | 6 [21] | **0.048** |
| Invasive ventilation before t=0 (days), median [Q1-Q3] | 2 [0-12] | 0 [0-6] | 0.239 |
| ^1^The ratio of antibiotic administration was calculated as the number of days with documented antibiotic use divided by 29, representing the proportion of the first 29 days exposed to antibiotics. ^2^The day on which the diagnostic work-up resulting in the LOS diagnosis was performed (and the corresponding matched day for the control infant) was defined as t=0. ^3^Full enteral feeding was defined as the first day on which both parenteral nutrition and intravenous glucose were ceased. ^4^Human milk was defined as mother’s own milk, donor human milk, or a combination. *Abbreviations: NICU = neonatal intensive care unit.* | | | |

**Table S8. Results of linear mixed-effects models assessing α-diversity per timeperiod.** α-diversity (Observed, Shannon index, and Chao1, respectively) was analysed using linear mixed-effects models to account for repeated measurements within individuals. Disease status, time period, and their interaction were included as fixed effects, with patient ID specified as a random intercept. Model-adjusted means with 95% confidence intervals were estimated using marginal means. Table displays regression coeffient/model estimate (β) and p-value for subgroup comparisons stratified per timeperiod (-10 to -9, -8 to -7, -6 to -4, and -3 to -1 days prior to LOS onset) in all cohorts (Discovery Cohort, Validation Cohort 1, and Validation Cohort 2, respectively).

| **Comparison** | **Metric** | **Timeperiod** | **Discovery cohort** | **Validation cohort 1** | **Validation cohort 2** |
| --- | --- | --- | --- | --- | --- |
| Overall LOS vs. controls | Observed | -1 to -3 days | β = 0.47, *p* = **0.001*** | β = 0.06, *p* = 0.728 | β = -0.09, *p* = 0.452 |
|  |  | -4 to -6 days | β = 0.49, *p* = **0.000*** | β = 0.24, *p* = 0.282 | β = -0.23, *p* = 0.072 |
|  |  | -7 to -8 days | β = 0.62, *p* = **0.000*** | NA | β = 0.02, *p* = 0.886 |
|  |  | -9 to -10 days | β = 0.26, *p* = 0.083 | NA | β = -0.15, *p* = 0.494 |
|  | Shannon | -1 to -3 days | β = 0.35, *p* = 0.063 | β = 0.05, *p* = 0.835 | β = -0.02, *p* = 0.859 |
|  |  | -4 to -6 days | β = 0.32, *p* = 0.075 | β = 0.41, *p* = 0.214 | β = 0.03, *p* = 0.820 |
|  |  | -7 to -8 days | β = 0.39, *p* = **0.048*** | NA | β = 0.25, *p* = 0.112 |
|  |  | -9 to -10 days | β = 0.33, *p* = 0.108 | NA | β = 0.26, *p* = 0.229 |
|  | Chao1 | -1 to -3 days | β = 0.40, *p* = **0.002*** | β = 0.11, *p* = 0.458 | β = -0.10, *p* = 0.393 |
|  |  | -4 to -6 days | β = 0.45, *p* = **0.001*** | β = 0.37, *p* = 0.089 | β = -0.21, *p* = 0.089 |
|  |  | -7 to -8 days | β = 0.55, *p* = 0.113 | NA | β = 0.04, *p* = 0.811 |
|  |  | -9 to -10 days | β = 0.26, *p* = **0.001*** | NA | β = -0.13, *p* = 0.542 |
| Non-staphylococcal LOS vs. controls | Observed | -1 to -3 days | β = 0.63, *p* = **0.000*** | β = 0.08, *p* = 0.689 | β = -0.09, *p* = 0.468 |
|  |  | -4 to -6 days | β = 0.50, *p* = **0.001*** | β = 0.15, *p* = 0.575 | β = -0.13, *p* = 0.316 |
|  |  | -7 to -8 days | β = 0.75, *p* = **0.000*** | NA | β = 0.14, *p* = 0.390 |
|  |  | -9 to -10 days | β = 0.40, *p* = **0.022*** | NA | β = 0.10, *p* = 0.673 |
|  | Shannon | -1 to -3 days | β = 0.49, *p* = **0.016*** | β = 0.05, *p* = 0.863 | β = -0.03, *p* = 0.840 |
|  |  | -4 to -6 days | β = 0.42, *p* = **0.032*** | β = 0.27, *p* = 0.486 | β = 0.05, *p* = 0.768 |
|  |  | -7 to -8 days | β = 0.58, *p* = **0.008*** | NA | β = 0.32, *p* = 0.087 |
|  |  | -9 to -10 days | β = 0.56, *p* = **0.016*** | NA | β = 0.22, *p* = 0.424 |
|  | Chao1 | -1 to -3 days | β = 0.56, *p* = **0.001*** | β = 0.15, *p* = 0.424 | β = -0.11, *p* = 0.344 |
|  |  | -4 to -6 days | β = 0.46, *p* = **0.004*** | β = 0.27, *p* = 0.285 | β = -0.12, *p* = 0.356 |
|  |  | -7 to -8 days | β = 0.71, *p* = **0.000*** | NA | β = 0.16, *p* = 0.329 |
|  |  | -9 to -10 days | β = 0.39, *p* = **0.032*** | NA | β = -0.10, *p* = 0.690 |
| *S. aureus*-LOS vs. Controls | Observed | -1 to -3 days | β = 0.00, *p* = 0.987 | β = 0.02, *p* = 0.923 | β = -0.11, *p* = 0.506 |
|  |  | -4 to -6 days | β = 0.52, *p* = **0.009*** | β = 0.43, *p* = 0.159 | β = -0.43, *p* = **0.025*** |
|  |  | -7 to -8 days | β = 0.41, *p* = **0.028*** | NA | β = -0.26, *p* = 0.240 |
|  |  | -9 to -10 days | β = 0.09, *p* = 0.640 | NA | β = -0.24, *p* = 0.412 |
|  | Shannon | -1 to -3 days | β = 0.09, *p* = 0.794 | β = 0.06, *p* = 0.862 | β = -0.02, *p* = 0.902 |
|  |  | -4 to -6 days | β = 0.16, *p* = 0.549 | β = 0.69, *p* = 0.179 | β = -0.00, *p* = 0.987 |
|  |  | -7 to -8 days | β = 0.11, *p* = 0.668 | NA | β = -0.11, *p* = 0.602 |
|  |  | -9 to -10 days | β = 0.03, *p* = 0.917 | NA | β = -0.32, *p* = 0.257 |
|  | Chao1 | -1 to -3 days | β = -0.03, *p* = 0.908 | β = 0.06, *p* = 0.776 | β = -0.09, *p* = 0.595 |
|  |  | -4 to -6 days | β = 0.51, *p* = **0.014*** | β = 0.58, *p* = **0.049*** | β = -0.41, *p* = **0.030*** |
|  |  | -7 to -8 days | β = 0.30, *p* = 0.121 | NA | β = -0.23, *p* = 0.281 |
|  |  | -9 to -10 days | β = 0.07, *p* = 0.725 | NA | β = -0.20, *p* = 0.487 |
| *E. coli*-LOS vs. controls | Observed | -1 to -3 days | β = 0.95, *p* = **0.000*** | β = 0.07, *p* = 0.720 | β = 0.08, *p* = 0.739 |
|  |  | -4 to -6 days | β = 0.63, *p* = **0.008*** | β = -0.01, *p* = 0.967 | β = 0.27, *p* = 0.314 |
|  |  | -7 to -8 days | β = 0.96, *p* = **0.000*** | NA | β = 0.32, *p* = 0.193 |
|  |  | -9 to -10 days | β = 0.47, *p* = **0.044*** | NA | β = 0.10, *p* = 0.765 |
|  | Shannon | -1 to -3 days | β = 0.52, *p* = **0.045*** | β = -0.09, *p* = 0.783 | β = 0.15, *p* = 0.610 |
|  |  | -4 to -6 days | β = 0.17, *p* = 0.585 | β = 0.20, *p* = 0.690 | β = 0.03, *p* = 0.919 |
|  |  | -7 to -8 days | β = 0.51, *p* = 0.094 | NA | β = 0.30, *p* = 0.312 |
|  |  | -9 to -10 days | β = 0.09, *p* = 0.760 | NA | β = 0.34, *p* = 0.394 |
|  | Chao1 | -1 to -3 days | β = 0.86, *p* = **0.000*** | β = 0.14, *p* = 0.464 | β = 0.06, *p* = 0.789 |
|  |  | -4 to -6 days | β = 0.58, *p* = **0.018*** | β = 0.16, *p* = 0.598 | β = -0.27, *p* = 0.306 |
|  |  | -7 to -8 days | β = 1.04, *p* = **0.000*** | NA | β = 0.35, *p* = 0.138 |
|  |  | -9 to -10 days | β = 0.50, *p* = **0.039*** | NA | β = 0.11, *p* = 0.724 |

**Table S9. Longitudinal differences in relative abundance of three top taxa between groups assessed with linear mixed-effects models.** Relative abundance was averaged per patient within each predefined time period (1-3 days, 4-6 days, 7-8 days and 9-10 days before late-onset sepsis). Disease status (case/control), time period (categorical), and their interaction were included as fixed effects, with patient ID as a random intercept. Model-adjusted means and 95% confidence intervals were estimated using marginal means. Results are shown only for taxa with at least one significant group comparison after False Discovery Rate (FDR) correction. Statistical significance was defined as p<0.05.

| **DISCOVERY COHORT** | | | | | | | |
| --- | --- | --- | --- | --- | --- | --- | --- |
| **Group** | **Taxon** | **Effect** | **Time period** | **Effect size (95% CI)** | **P-value** | **p_FDR** | **Direction** |
| ***S. aureus* LOS** | g_Staphylococcus spp. | Group |  |  | 0.460 | 0.919 |  |
|  |  | Group x Period |  |  | **0.01** | 0.199 |  |
|  |  | Period |  |  | 0.047 | 0.14 |  |
|  |  | Between-group difference | -9 to -10 days | 0.39 (0.15-0.62) | **0.002** | **0.016** | ↑ |
|  |  |  | -7 to -8 days | 0.04 (-0.19-0.27) | 0.731 | 0.874 | ↑ |
|  |  |  | -4 to -6 days | -0.14 (-0.4-0.12) | 0.282 | 0.627 | ↓ |
|  |  |  | -1 to -3 days | -0.06 (-0.34-0.22) | 0.662 | 0.836 | ↓ |
| **Non-staphylococcal LOS** | g_Escherichia-Shigella | Group |  |  | **0.001** | **0.004** |  |
|  |  | Group x Period |  |  | 0.365 | 0.576 |  |
|  |  | Period |  |  | 0.299 | 0.359 |  |
|  |  | Between-group difference | -9 to -10 days | 0.39 (0.13-0.64) | **0.004** | **0.029** | ↑ |
|  |  |  | -7 to -8 days | 0.53 (0.28-0.78) | **<0.001** | **0.001** | ↑ |
|  |  |  | -4 to -6 days | 0.4 (0.15-0.64) | **0.002** | **0.018** | ↑ |
|  |  |  | -1 to -3 days | 0.36 (0.11-0.62) | **0.006** | **0.036** | ↑ |
| ***E. coli* LOS** | g_Escherichia-Shigella | Group |  |  | **<0.001** | **<0.001** |  |
|  |  | Group x Period |  |  | 0.157 | 0.47 |  |
|  |  | Period |  |  | **0.041** | 0.14 |  |
|  |  | Between-group difference | -9 to -10 days | 0.44 (0.12-0.76) | **0.009** | **0.046** | ↑ |
|  |  |  | -7 to -8 days | 0.74 (0.42-1.07) | **<0.001** | **0.001** | ↑ |
|  |  |  | -4 to -6 days | 0.73 (0.42-1.03) | **<0.001** | **0.001** | ↑ |
|  |  |  | -1 to -3 days | 0.7 (0.39-1) | **<0.001** | **0.001** | ↑ |
| **VALIDATION COHORT 1** | | | | | | | |
| **Non-staphylococcal LOS** | g_Escherichia-Shigella | Group |  |  | **0.004** | **0.044** |  |
|  |  | Group x Period |  |  | 0.192 | 0.767 |  |
|  |  | Period |  |  | 0.085 | 0.17 |  |
|  |  | Between-group difference | -4 to -5 days | 0.39 (0.08-0.7) | **0.016** | 0.102 | ↑ |
|  |  |  | -1 to -3 days | 0.51 (0.22-0.8) | **0.002** | **0.019** | ↑ |
| ***E. coli* LOS** | g_Escherichia-Shigella | Group |  |  | **0.009** | **0.044** |  |
|  |  | Group x Period |  |  | **0.003** | **0.041** |  |
|  |  | Period |  |  | **0.002** | **0.016** |  |
|  |  | Between-group difference | -4 to -5 days | 0.2 (-0.09-0.49) | 0.159 | 0.545 | ↑ |
|  |  |  | -1 to -3 days | 0.55 (0.29-0.82) | **<0.001** | **0.011** | ↑ |
| ***E. coli* LOS** | g_Staphylococcus spp. | Group |  |  | **0.011** | **0.044** |  |
|  |  | Group x Period |  |  | **0.028** | 0.167 |  |
|  |  | Period |  |  | **0.003** | **0.016** |  |
|  |  | Between-group difference | -4 to -5 days | 0.69 (0.14-1.24) | **0.017** | 0.102 | ↑ |
|  |  |  | -1 to -3 days | 0.08 (-0.17-0.32) | 0.530 | 0.882 | ↑ |
| **VALIDATION COHORT 2** | | | | | | | |
| ***E. coli* LOS** | g_Escherichia-Shigella | Group |  |  | **<0.001** | **0.004** |  |
|  |  | Group x Period |  |  | **0.047** | 0.562 |  |
|  |  | Period |  |  | **0.042** | 0.1 |  |
|  |  | Between-group difference | -9 to -10 days | 0.81 (0.27-1.35) | **0.004** | 0.103 | ↑ |
|  |  |  | -7 to -8 days | 1.09 (0.49-1.69) | **0.001** | **0.028** | ↑ |
|  |  |  | -4 to -6 days | 0.23 (-0.26-0.71) | 0.353 | 0.769 | ↑ |
|  |  |  | -1 to -3 days | 0.62 (0.19-1.06) | **0.006** | 0.103 | ↑ |

**Table S10. Significant discriminating microbiota features identified by MaAsLin2 across all three cohorts.** The table shows taxa at the genus, family, and order level that discriminated between disease and control groups. Features were included only if they were represented by ≥2 significant Amplicon Sequencing Variants (ASVs) (q-value <0.05). The arrows indicate the direction of the coefficient for the reference group relative to the other group; only positive (⬆), only negative (⬇), or both (⬍). There were no discriminating features discovered by MaAsLin2 analysis in VC1 and VC2.

| **Comparisons** Reference group vs. other group | | **Discovery Cohort** | **Validation Cohort 1** | **Validation Cohort 2** |
| --- | --- | --- | --- | --- |
|  |  | **Microbiota feature (number of discriminating ASVs)** | | |
| **Different ASVs total** | | **76** | **0** | **0** |
| Overall LOS | Controls | f_Enterobacteriaceae (24) ⬆ | n.a. | n.a. |
|  |  | g_Escherichia-Shigella (10) ⬆ |  |  |
|  |  | f_Yersiniaceae (7) ⬍ |  |  |
|  |  | o_Enterobacterales (6) ⬆ |  |  |
|  |  | g_Citrobacter (4) ⬆ |  |  |
|  |  | g_Serratia (4) ⬍ |  |  |
|  |  | g_Yersinia (4) ⬆ |  |  |
|  |  | g_Bacteroides (2) ⬍ |  |  |
|  |  | g_Cedecea (2) ⬆ |  |  |
|  |  | g_Raoultella (2) ⬆ |  |  |
|  |  | f_Comamonadaceae (2) ⬇ |  |  |
| **Different ASVs total** | | **155** | **0** | **0** |
| Non-staphylococcal LOS | Controls | f_Enterobacteriaceae (46) ⬆ | n.a. | n.a. |
|  |  | g_Escherichia-Shigella (41) ⬆ |  |  |
|  |  | f_Yersiniaceae (15) ⬆ |  |  |
|  |  | o_Enterobacterales (8) ⬆ |  |  |
|  |  | g_Yersinia (7) ⬆ |  |  |
|  |  | g_Serratia (5) ⬍ |  |  |
|  |  | g_Cedecea (4) ⬆ |  |  |
|  |  | g_Citrobacter (4) ⬆ |  |  |
|  |  | g_Leuconostoc (3) ⬆ |  |  |
|  |  | g_Raoultella (3) ⬆ |  |  |
|  |  | g_Bacteroides (2) ⬍ |  |  |
|  |  | g_Faecalibacterium (2) ⬆ |  |  |
|  |  | g_Ralstonia (2) ⬇ |  |  |
|  |  | f_Comamonadaceae (2) ⬇ |  |  |
| **Different ASVs total** | | **25** | **0** | **0** |
| *S. aureus*-LOS | Controls | g_Yersinia (4) ⬆ | n.a. | n.a. |
|  |  | f_Yersiniaceae (4) ⬆ |  |  |
|  |  | g_Ralstonia (2) ⬇ |  |  |
|  |  | g_Serratia (2) ⬍ |  |  |
|  |  | f_Comamonadaceae (2) ⬇ |  |  |
|  |  | f_Enterobacteriaceae (2)⬆ |  |  |
|  |  | o_Enterobacterales (2) ⬆ |  |  |
| **Different ASVs total** | | **3** | **0** | **0** |
| *S. aureus*-LOS | non-staphylococcal LOS | g_Escherichia-Shigella (3) ⬇ | n.a. | n.a. |
| **Different ASVs total** | | **125** | **0** | **0** |
| *E. coli*-LOS | Controls | g_Escherichia-Shigella (51) ⬆ | n.a. | n.a. |
|  |  | f_Enterobacteriaceae (36) ⬆ |  |  |
|  |  | f_Yersiniaceae (11) ⬆ |  |  |
|  |  | g_Yersinia (5) ⬆ |  |  |
|  |  | g_Serratia (4) ⬍ |  |  |
|  |  | o_Enterobacterales (4) ⬆ |  |  |
|  |  | g_Bifidobacterium (2) ⬆ |  |  |
|  |  | g_Raoultella (2) ⬆ |  |  |

**Table S11. Top 20 ASVs identified by the random forest machine learning model as the most important microbial features contributing to the early late-onset sepsis risk stratification for non-staphylococcal late-onset sepsis versus controls.** Table displays the ranking of the top 20 ASVs contributing most to the predictive performance of the Random Forest model. For each ASV, the corresponding taxonomic classification (family, genus, and species) and its importance score within the model are presented. Variable importance scores are unitless and scaled to 100 for the top feature to represent relative contributions. Species-level assignments should be interpreted with caution due to the limited taxonomic resolution of 16S rRNA V3–V4 sequencing.

| **Rank** | **Family** | **Genus** | **Species** | **Importance** |
| --- | --- | --- | --- | --- |
| **1** | Bacteroidaceae | *Bacteroides* | *fragilis* | 100 |
| **2** | Enterococcaceae | *Enterococcus* | *faecium* | 43 |
| **3** | Erwiniaceae | *Pantoea* | *ananatis* | 34 |
| **4** | Staphylococcaceae | *Staphylococcus* | *haemolyticus* | 29 |
| **5** | Bifidobacteriaceae | *Bifidobacterium* | *breve* | 26 |
| **6** | Veillonellaceae | *Veillonella* | *dispar* | 23 |
| **7** | Staphylococcaceae | *Staphylococcus* | *aureus* | 21 |
| **8** | Bacteroidaceae | *Bacteroides* | *vulgatus* | 15 |
| **9** | Streptococcaceae | *Streptococcus* | *anginosus* | 13 |
| **10** | Veillonellaceae | *Veillonella* | *parvula* | 13 |
| **11** | Staphylococcaceae | *Staphylococcus* | *warneri* | 13 |
| **12** | Bacteroidaceae | *Bacteroides* | *dorei* | 12 |
| **13** | Staphylococcaceae | *Staphylococcus* | *auricularis* | 11 |
| **14** | Lysobacteraceae | *Stenotrophomonas* | *maltophilia* | 11 |
| **15** | Clostridiaceae | *Clostridium* | *perfringens* | 9 |
| **16** | Corynebacteriaceae | *Corynebacterium* | *tuberculostearicum* | 7 |
| **17** | Streptococcaceae | *Streptococcus* | *agalactiae* | 5 |
| **18** | Corynebacteriaceae | *Corynebacterium* | *kroppenstedtii* | 5 |
| **19** | Pasteurellaceae | *Haemophilus* | *parainfluenzae* | 5 |
| **20** | Actinomycetaceae | *Winkia* | *neuii* | 3 |

**Table S12. Top 20 ASVs identified by the random forest machine learning model as the most important microbial features contributing to the early late-onset sepsis risk stratification for *E. coli*-LOS versus controls.** Table displays the ranking of the top 20 ASVs contributing most to the predictive performance of the Random Forest model. For each ASV, the corresponding taxonomic classification (family, genus, and species) and its importance score within the model are presented. Variable importance scores (mean decrease in Gini index) are unitless and scaled to 100 for the top feature to represent relative contributions. Species-level assignments should be interpreted with caution due to the limited taxonomic resolution of 16S rRNA V3–V4 sequencing.

| **Rank** | **Family** | **Genus** | **Species** | **Importance** |
| --- | --- | --- | --- | --- |
| **1** | Yersiniaceae | *Serratia* | NA | 100 |
| **2** | Yersiniaceae | *Yersinia* | NA | 79 |
| **3** | Enterobacteriaceae | *Escherichia-Shigella* | NA | 70 |
| **4** | Enterobacteriaceae | *Escherichia-Shigella* | NA | 51 |
| **5** | Enterobacteriaceae | *Klebsiella* | NA | 41 |
| **6** | Enterobacteriaceae | *Escherichia-Shigella* | NA | 30 |
| **7** | Bacteroidaceae | *Bacteroides* | *fragilis* | 28 |
| **8** | Bifidobacteriaceae | *Bifidobacterium* | *breve* | 24 |
| **9** | Enterobacteriaceae | *Klebsiella* | NA | 16 |
| **10** | Erwiniaceae | *Pantoea* | *ananatis* | 15 |
| **11** | Veillonellaceae | *Veillonella* | NA | 12 |
| **12** | Streptococcaceae | *Streptococcus* | *agalactiae* | 11 |
| **13** | Veillonellaceae | *Veilonella* | *dispar* | 11 |
| **14** | Enterococcaceae | *Enterococcus* | *faecium* | 8 |
| **15** | Enterobacteriaceae | *Citrobacter* | NA | 6 |
| **16** | Staphylococcaceae | *Staphylococcus* | *aureus* | 5 |
| **17** | Staphylococcaceae | *Staphylococcus* | NA | 5 |
| **18** | Enterobacteriaceae | *Citrobacter* | NA | 5 |
| **19** | Enterobacteriaceae | *Salmonella* | NA | 5 |
| **20** | Enterobacteriaceae | *Citrobacter* | NA | 5 |

**SUPPLEMENTARY FIGURES**

**
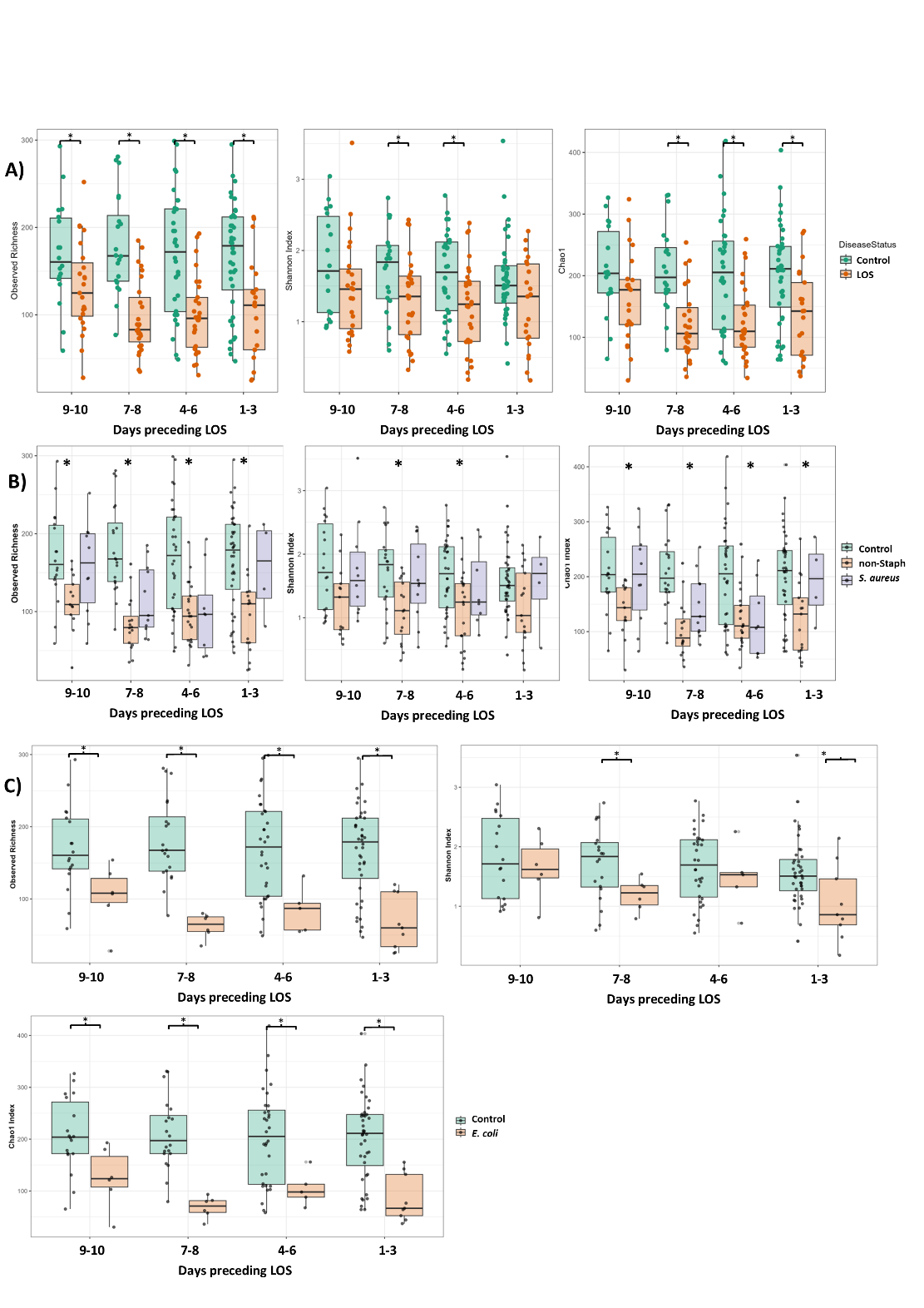
**

**Figure S1. α-diversity in fecal samples up to 10 days prior to clinical onset of late-onset sepsis versus controls in the Discovery Cohort.** The boxplots display the distribution of three α-diversity metrics (Observed Richness, Shannon index, and Chao1, respectively) over a longitudinal course preceding LOS for **A)** infants with LOS versus controls, **B)** infants with *S. aureus*-LOS, non-staphylococcal LOS, and controls, and **C)** *E. coli*-LOS versus controls. This figure demonstrates a consistent decrease in α-diversity over a period of 10 days before LOS compared to controls in preterm infants in the Discovery Cohort.*A p-value<0.05, as assessed by a Wilcoxon Rank-Sum or Kruskal-Wallis test, was considered significant. Abbreviations: LOS, late-onset sepsis.


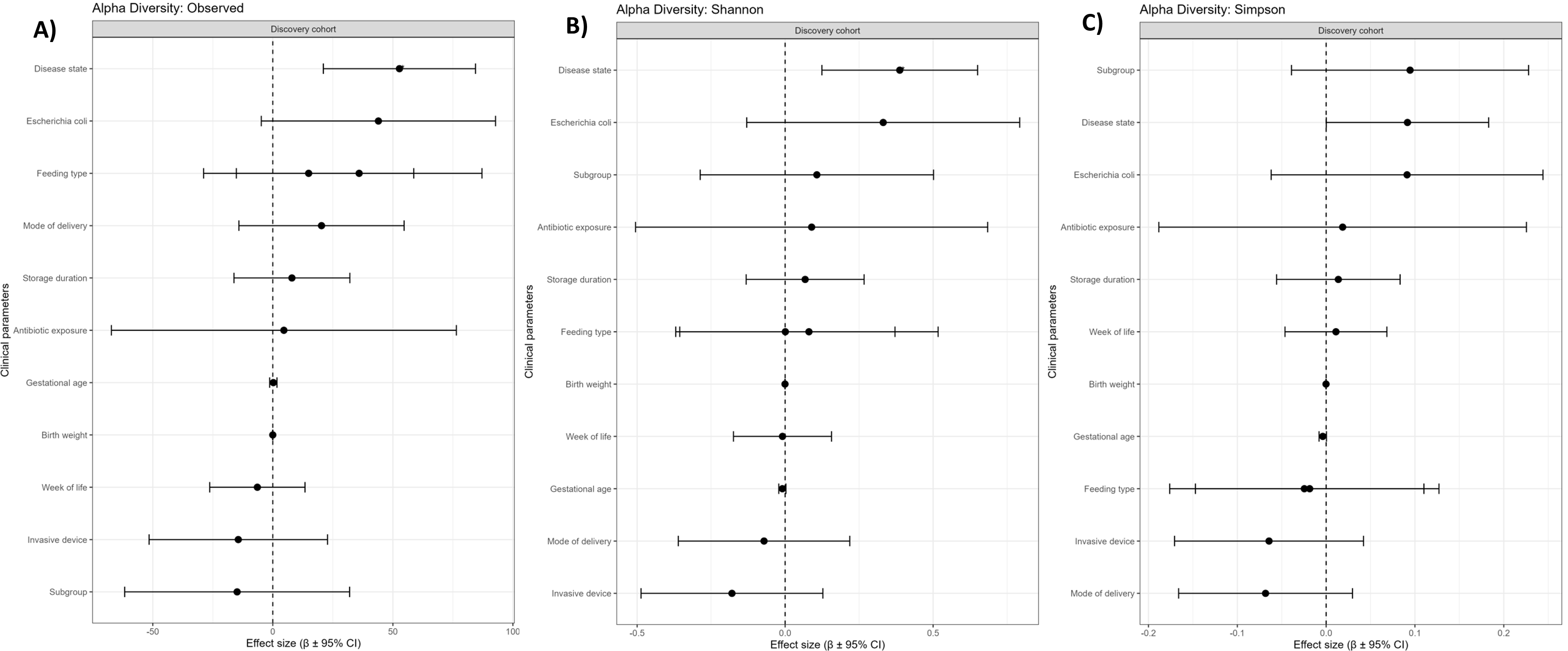


**Figure S2. Associations between clinical factors and α-diversity indices in the Discovery Cohort.** Associations are assessed using univariate linear mixed-effects models. Effect sizes are presented as β coefficients with 95% confidence intervals (CIs) for the A) Observed, B) Shannon, and C) Simpson diversity metrics. Apart from a significant association with disease state for the Observed and Shannon metrics, no other clinical factors showed significant associations.


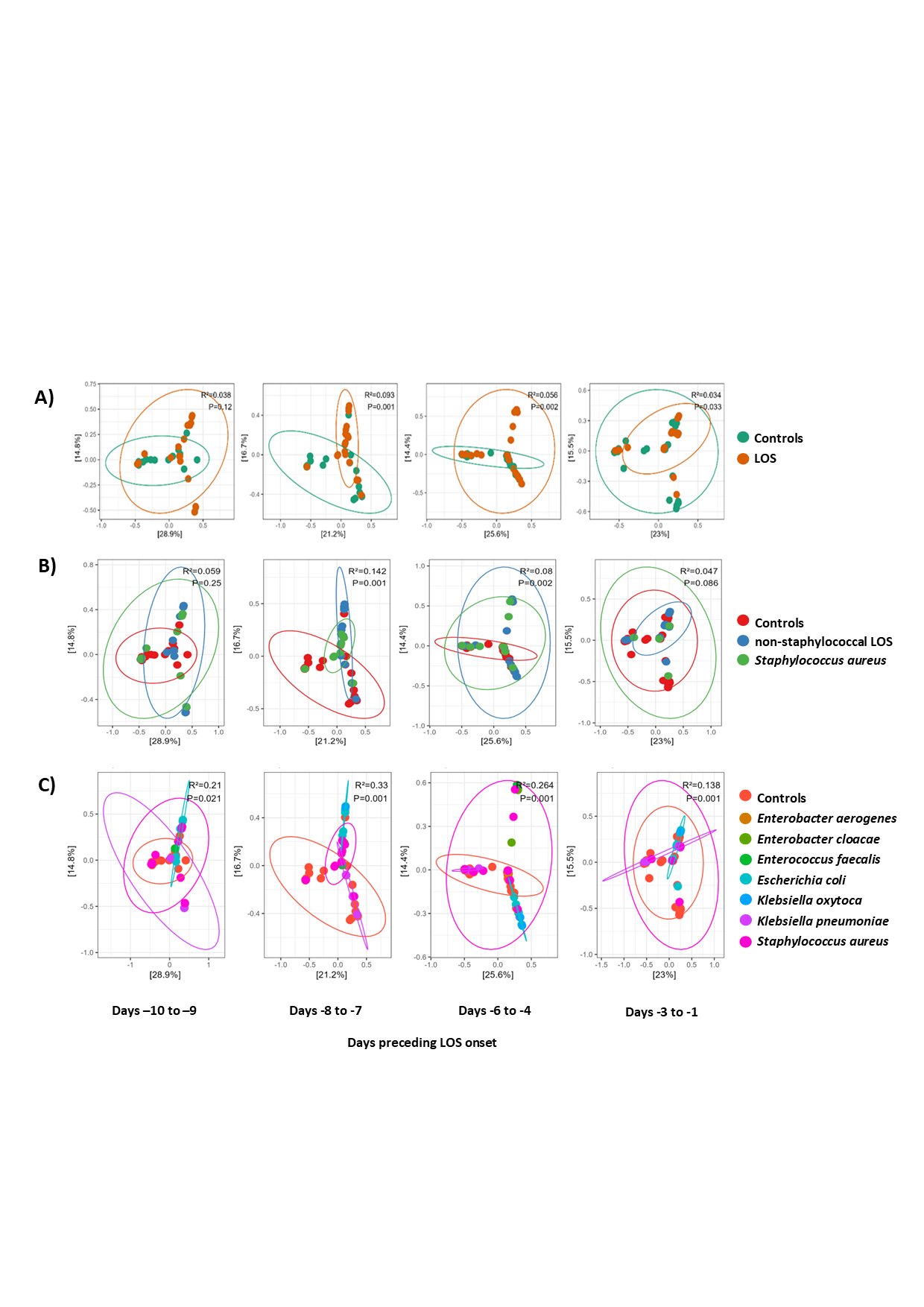


**Figure S3.  β-diversity in fecal samples up to 10 days prior to clinical onset of late-onset sepsis versus controls per time period in the Discovery Cohort.** Bacterial β-diversity as assessed by PCoA based on Bray-Curtis dissimilarity is displayed. Potential clustering of fecal samples based on microbiome composition is assessed for all infants per time period (-10 to -9, -8 to -7, -6 to -4, and -3 to -1 days prior to LOS onset) in **A)** infants with LOS versus controls, **B)** infants with *S. aureus*-LOS, non-staphylococcal LOS, and controls, and **C)** *E. coli*-LOS versus controls. Statistical analysis was performed by PERMANOVA. A p-value below 0.05 was considered significant.

**
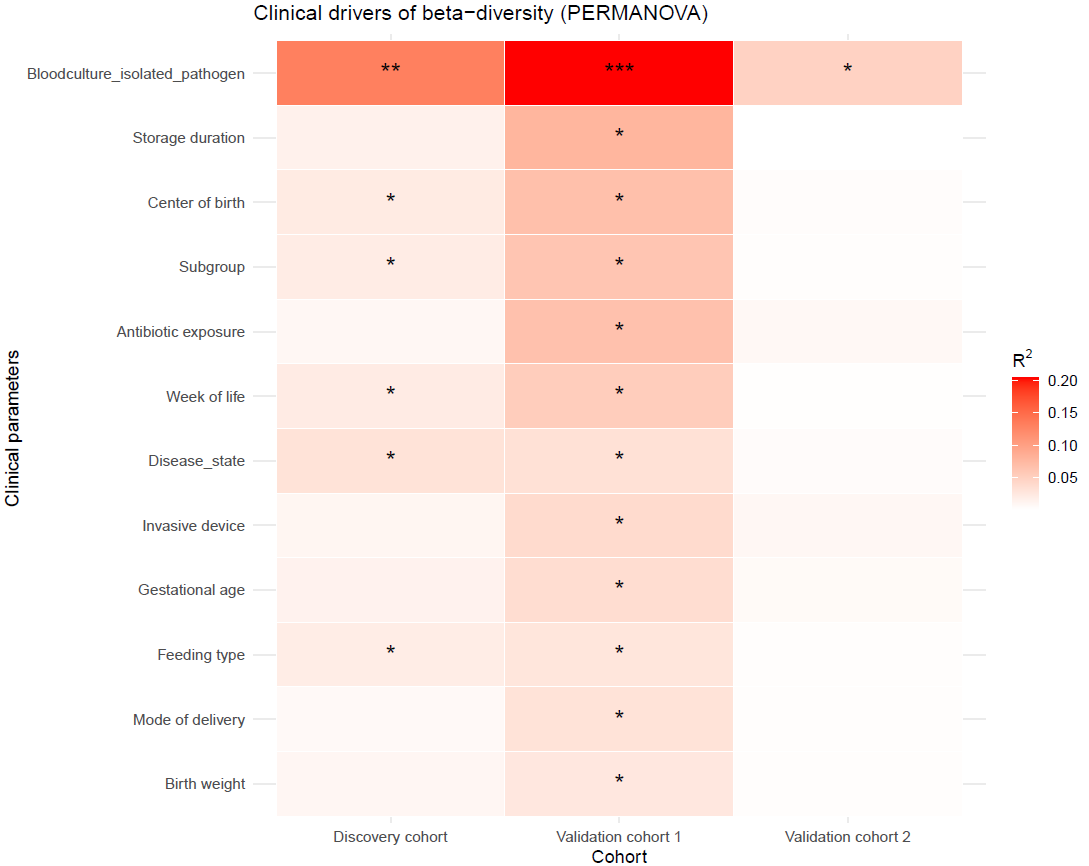
**

**Figure S4. Heatmap of clinical drivers of β-diversity across all cohorts.** Associations between key clinical parameters and β-diversity were assessed using PERMANOVA analyses across the Discovery cohort and Validation cohorts 1 and 2. Results are presented as a heatmap, with colors representing the proportion of variance explained (R²) by each clinical parameter. Analyses were corrected for repeated measurements by accounting for patient ID. Statistical significance is indicated by asterisk. For all three cohorts, the blood culture isolated pathogen is the strongest driver of β-diversity.

**
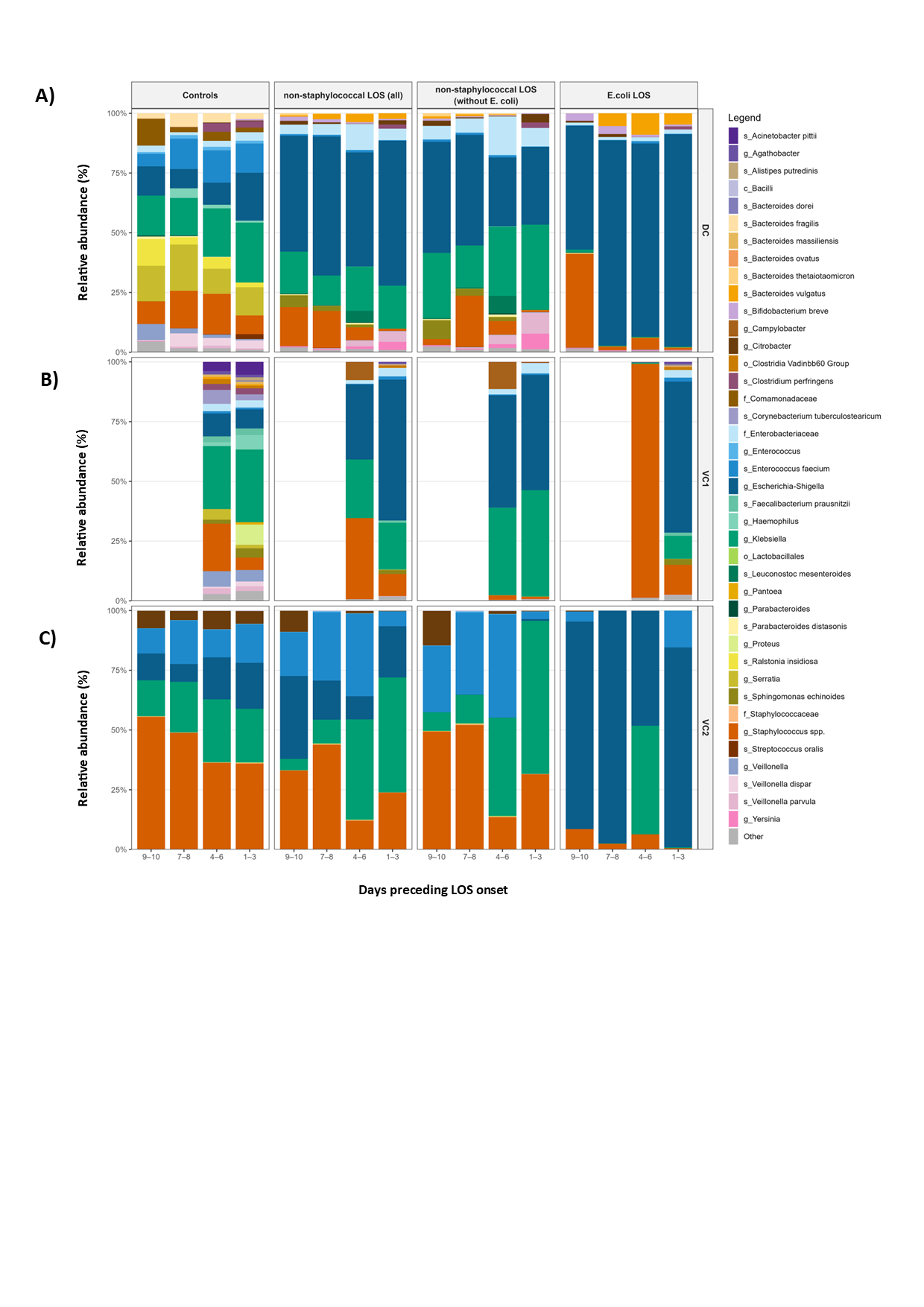
**

**Figure S5. Temporal dynamics of gut microbiota composition at the genus level during the ten-day window preceding clinical onset of late-onset sepsis (LOS) of infants with non-staphylococcal late-onset sepsis (LOS) with and without infants with *E.coli* LOS, and controls in the Discovery Cohort (DC) and Validation Cohorts 1 and 2 (VC1/VC2)**. Stacked bar plots displaying the mean relative abundance of the 25 most abundant bacterial species and/or genera across four time periods (9–10, 7–8, 4–6, 1–3 days before LOS). In this figure the non-staphylococcal LOS group is divided into two subgroups. The panels show the following from left to right: 1) Controls, 2) LOS cases caused by all blood culture–isolated pathogens within the non-staphylococcal LOS group (non-staphylococcal LOS (all), 3) the non-staphylococcal LOS group excluding samples from infants with *E.coli-*LOS, and 4) infants with *E.coli-*LOS. This separation of the non-staphylococcal group allows us to determine whether the dominant presence of *Escherichia/Shigella* observed in the non-staphylococcal LOS group is primarily driven by *E. coli*-LOS cases, or if *Escherichia/Shigella* also represents a predominant feature of the microbiota profiles in infants with LOS caused by the other non-staphylococcal pathogens. To avoid overinterpretation of species-level assignments from 16S rRNA sequencing, *Staphylococcus* ASVs were collapsed and reported as *Staphylococcus* spp.


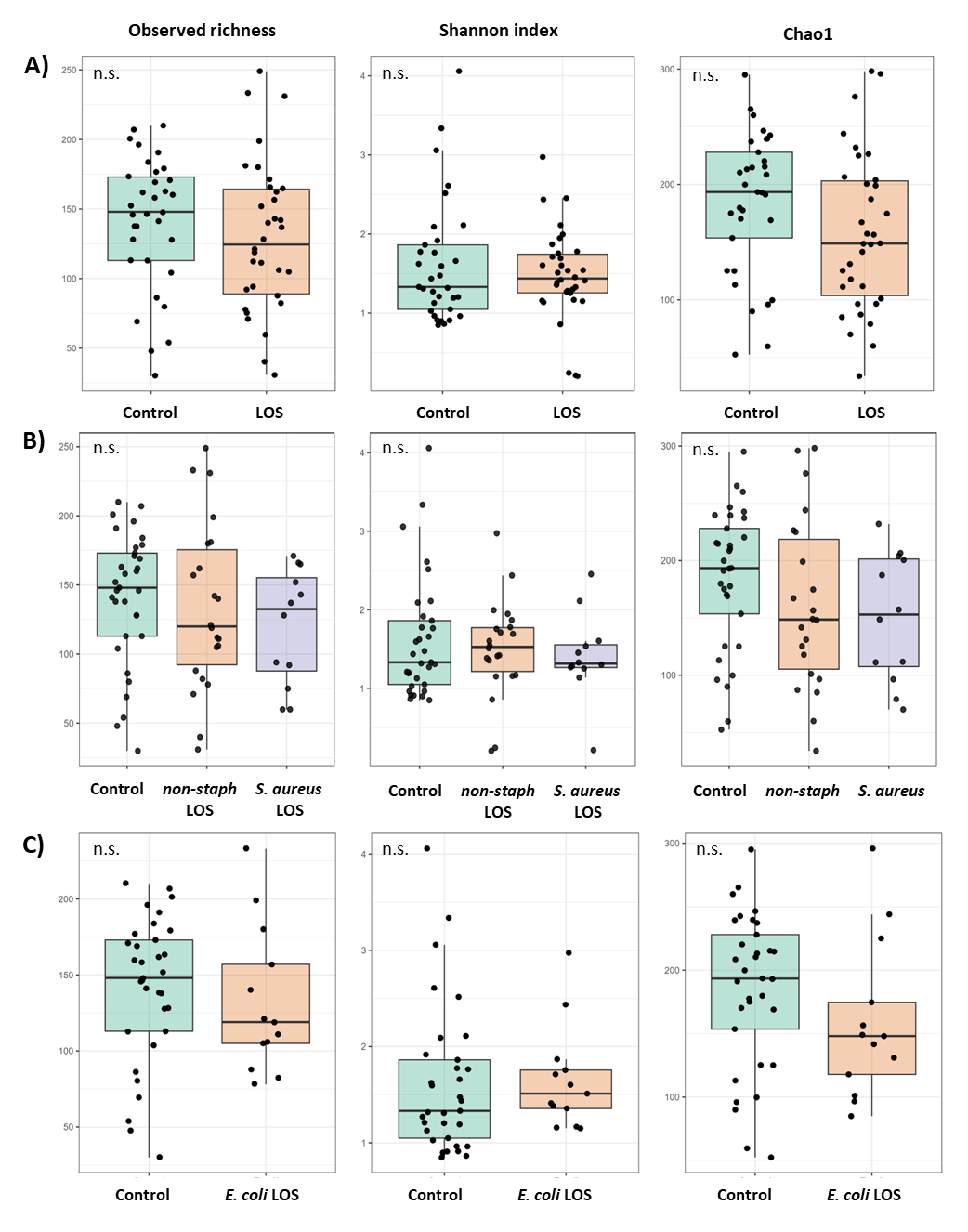


**Figure S6. α-diversity in fecal samples up to 10 days prior to clinical onset of late-onset sepsis versus controls in the Validation Cohort 1 (VC1).** The boxplots display the distribution of three α-diversity metrics (Observed Richness, Shannon index, and Chao1, respectively) over a longitudinal course preceding LOS for **A)** infants with LOS versus controls, **B)** infants with *S. aureus*-LOS, non-staphylococcal LOS, and controls, and **C)** *E. coli*-LOS versus controls. In none of the subgroups there is a significant difference demonstrated in α-diversity in Validation Cohort 1. *A p-value<0.05, as assessed by a Wilcoxon Rank-Sum or Kruskal-Wallis test, was considered significant. *Abbreviations: LOS, late-onset sepsis; non-staph, non-staphylococcal.*


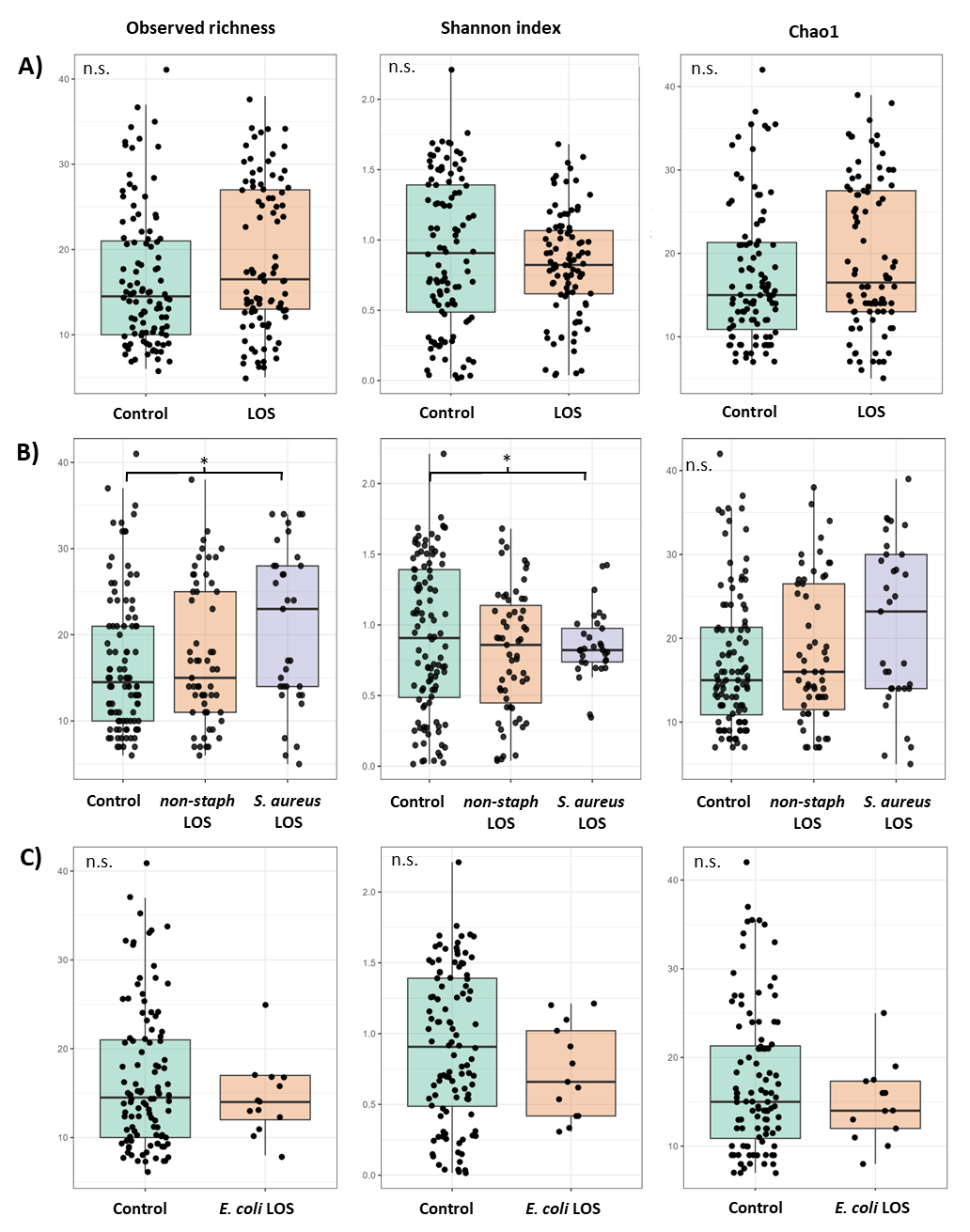


**Figure S7. α-diversity in fecal samples up to 10 days prior to clinical onset of late-onset sepsis versus controls in the Validation Cohort 2 (VC2).** The boxplots display the distribution of three α-diversity metrics (Observed Richness, Shannon index, and Chao1, respectively) over a longitudinal course preceding LOS for **A)** infants with LOS versus controls, **B)** infants with *S. aureus*-LOS, non-staphylococcal LOS, and controls, and **C)** *E. coli*-LOS versus controls. α-diversity was only significantly different for controls vs. *S. aureus*-LOS (Observed and Shannon). A p-value<0.05, as assessed by a Wilcoxon Rank-Sum or Kruskal-Wallis test, was considered significant. *Abbreviations: LOS, late-onset sepsis; non-staph, non-staphylococcal.*


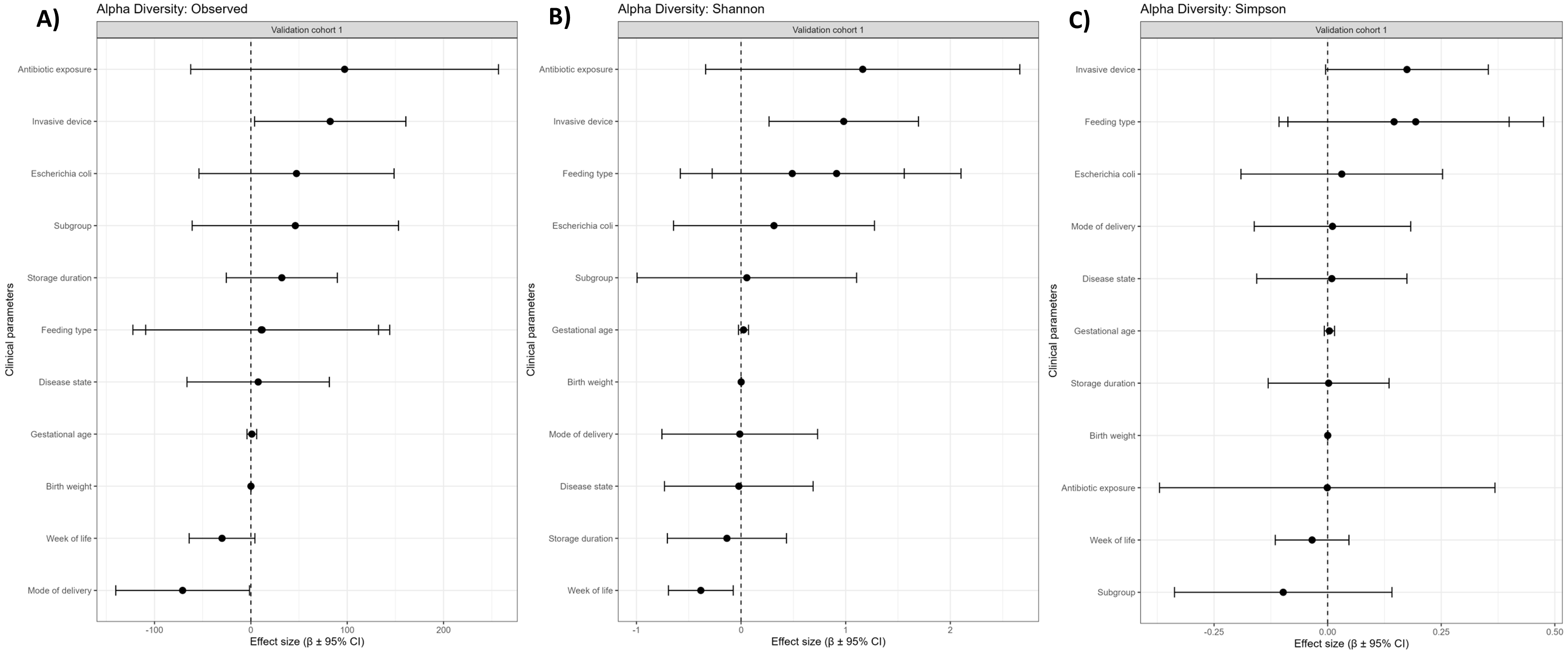


**Figure S8. Associations between clinical factors and α-diversity indices in Validation Cohort 1.** Associations are assessed using univariate linear mixed-effects models. Effect sizes are presented as β coefficients with 95% confidence intervals (CIs) for the A) Observed, B) Shannon, and C) Simpson diversity metrics. Apart from a significant association with having an invasive device before late-onset sepsis for the Observed and Shannon metrics, mode of delivery for Observed, and sample week of life for the Shannon metric, no other clinical factors showed significant associations.


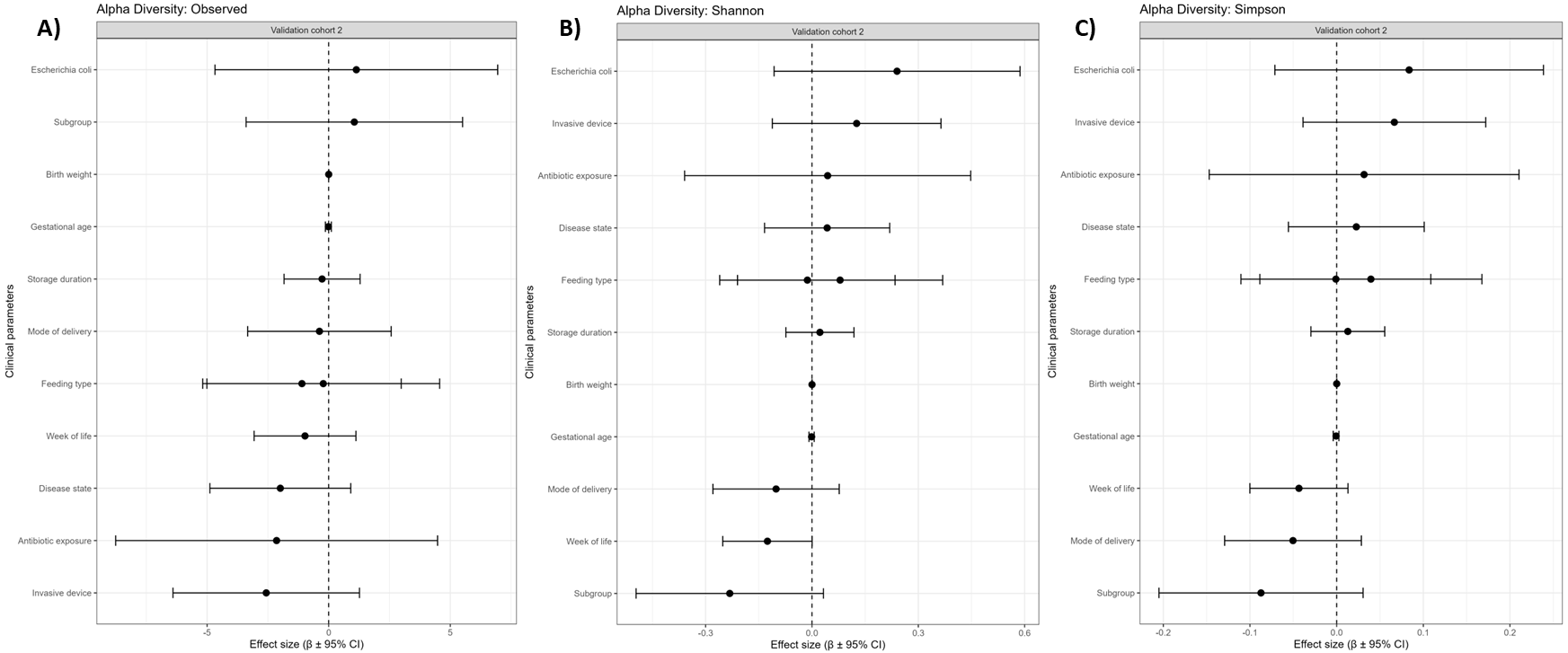


**Figure S9. Associations between clinical factors and α-diversity indices in Validation Cohort 2.** Associations are assessed using univariate linear mixed-effects models. Effect sizes are presented as β coefficients with 95% confidence intervals (CIs) for the A) Observed, B) Shannon, and C) Simpson diversity metrics. No other clinical factors showed significant associations.


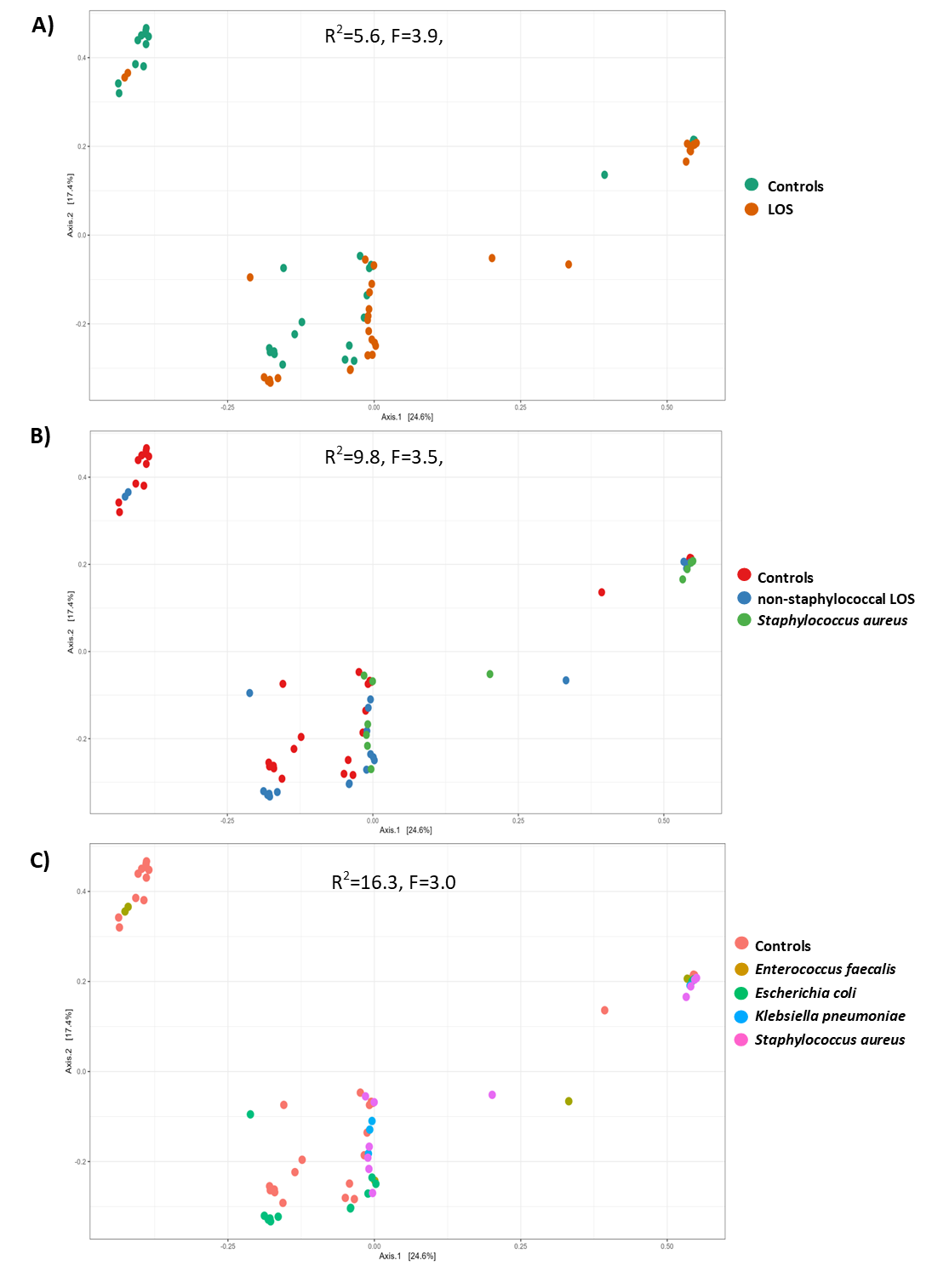


**Figure S10. β-diversity in fecal samples up to 5 days prior to clinical onset of late-onset sepsis versus controls for Validation Cohort 1.** Bacterial β-diversity as assessed by PCoA based on Bray-Curtis dissimilarity is displayed. Potential clustering of fecal samples based on microbiome composition is assessed for **A)** all infants with LOS versus healthy controls, **B)** *S. aureus*-LOS, non-staphylococcal LOS, and controls, and **C)** LOS by specific causative pathogens (blood culture isolated pathogen) versus controls. All data was normalized before assessment. Statistical analysis was performed by PERMANOVA. A p-value below 0.05 was considered significant. Similar to the Discovery Cohort, the figure demonstrates that the largest variation in β-diversity is explained by type of causative pathogen (**C**, R^2^=16.3%, F=3.0, p=0.001), followed by type of sepsis (**B**, R^2^=9.8%, F=3.5, p=0.001), and least variation is explained by disease state (**A**, LOS vs. controls, R^2^=5.6%, F=3.9, p=0.001) in Validation Cohort 1. *Abbreviations: LOS, late-onset sepsis; non-staph, non-staphylococcal.*


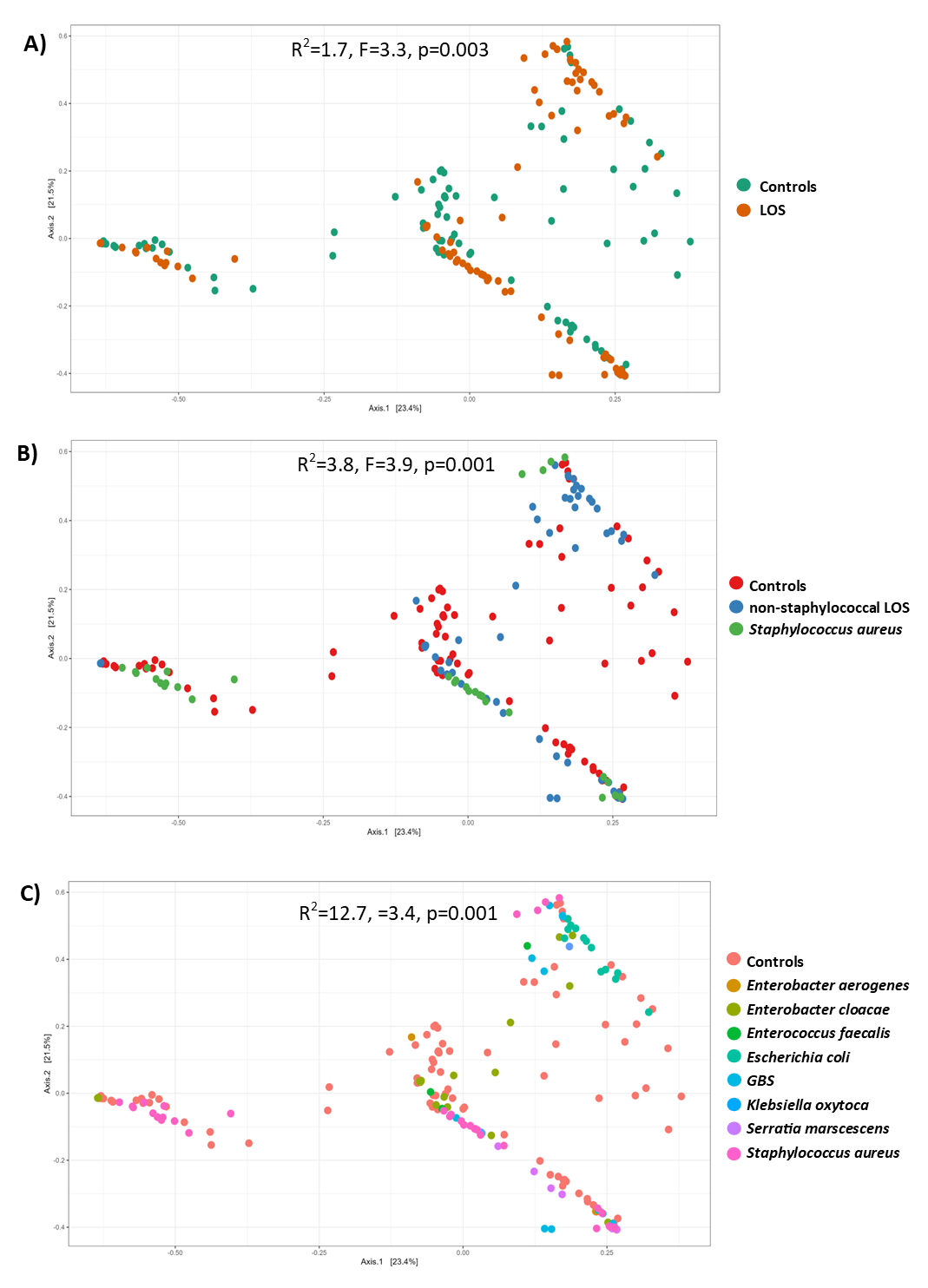


**Figure S11.  β-diversity in fecal samples up to 10 days prior to clinical onset of late-onset sepsis versus controls for Validation Cohort 2.** Bacterial β-diversity as assessed by PCoA based on Bray-Curtis dissimilarity is displayed. Potential clustering of fecal samples based on microbiome composition is assessed for **A)** all infants with LOS versus healthy controls, **B)** *S. aureus*-LOS, non-staphylococcal LOS, and controls, and **C)** LOS by specific causative pathogens (blood culture isolated pathogen) versus controls. All data was normalized before assessment. Statistical analysis was performed by PERMANOVA. A p-value below 0.05 was considered significant. Similar to the Discovery Cohort and Validation Cohort 1, the figure demonstrates that the largest variation in β-diversity is explained by type of causative pathogen (**C**, R^2^=12.7%, F=3.4, p=0.001). Minimal variation is explained by type of sepsis (**B**, R^2^=3.8%, F=3.9, p=0.001), and disease state (**A**, LOS vs. controls, R^2^=1.7%, F=3.3, p=0.003) in Validation Cohort 2. *Abbreviations: LOS, late-onset sepsis; non-staph, non-staphylococcal.*

**
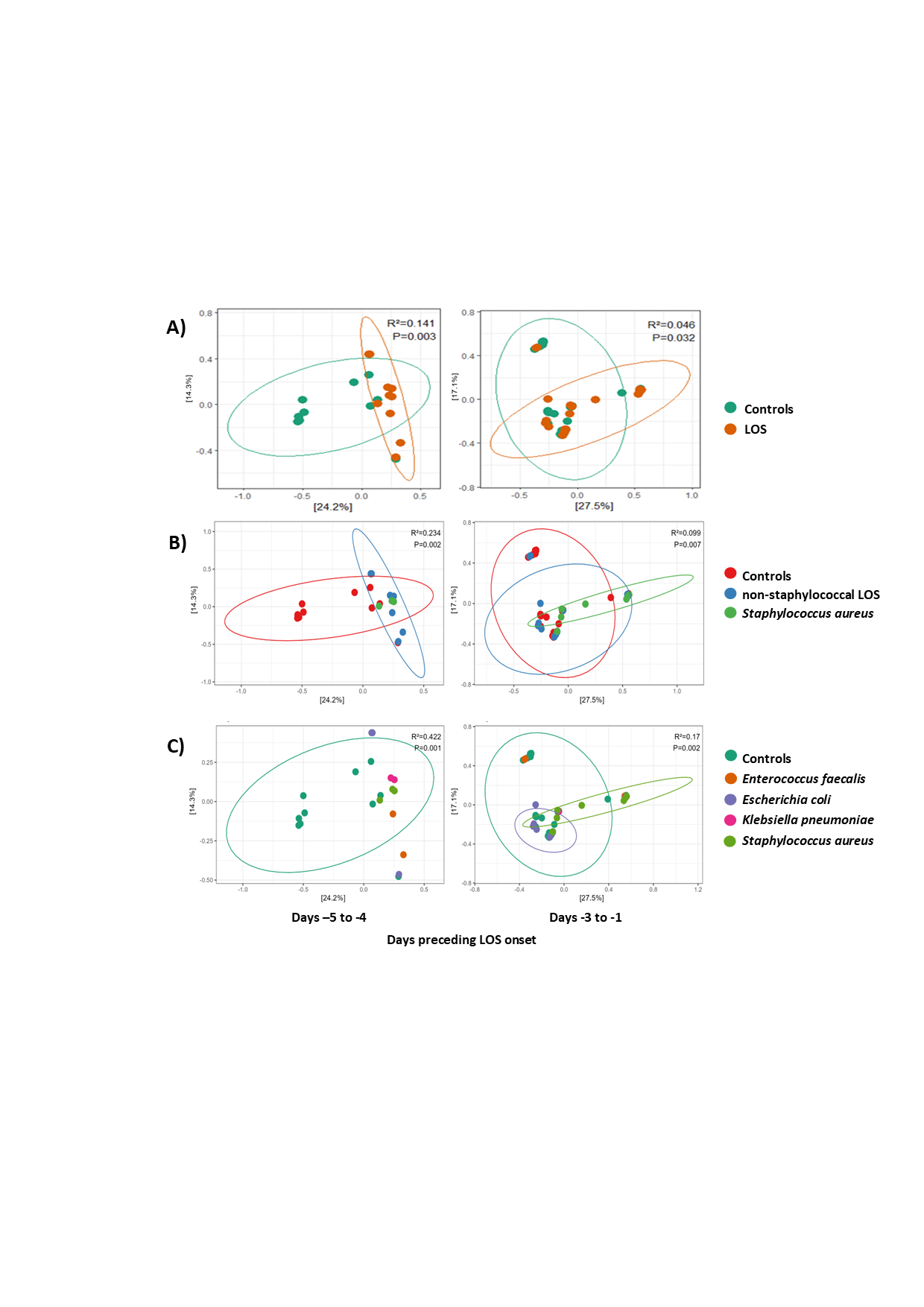
**

**Figure S12:  β-diversity in fecal samples up to 10 days prior to clinical onset of late-onset sepsis versus controls per time period in Validation Cohort 1.** Bacterial β-diversity as assessed by PCoA based on Bray-Curtis dissimilarity is displayed. Potential clustering of fecal samples based on microbiome composition is assessed for all infants per time period (-10 to -9, -8 to -7, -6 to -4, and -3 to -1 days prior to LOS onset) in **A)** infants with LOS versus controls, **B)** infants with *S. aureus*-LOS, non-staphylococcal LOS, and controls, and **C)** *E. coli*-LOS versus controls. Only one sample per infant (closest to LOS) per time period was analyzed to account for repeated measures. Statistical analysis was performed by PERMANOVA. A p-value below 0.05 was considered significant.

**
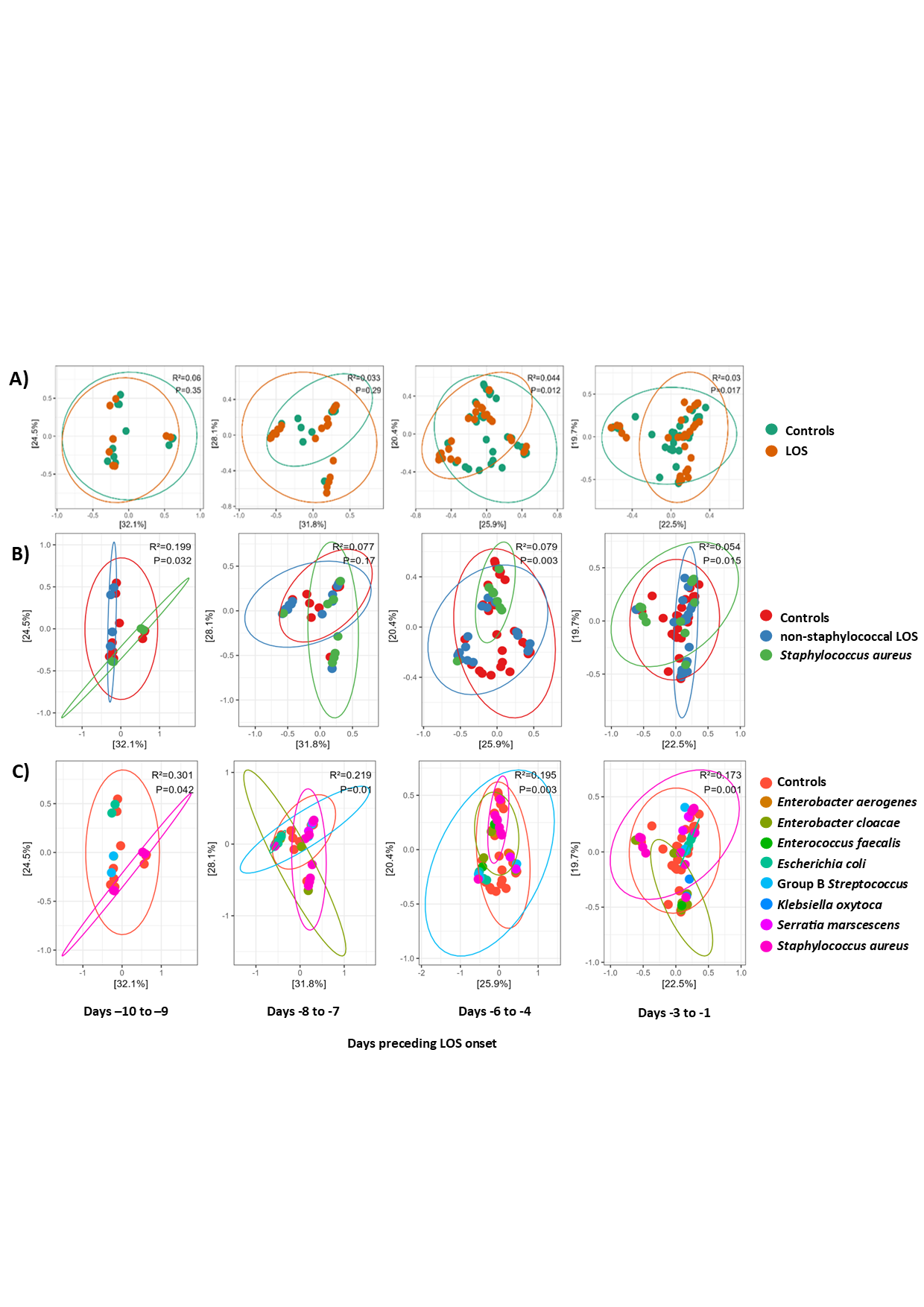
Figure S13. β-diversity in fecal samples up to 10 days prior to clinical onset of late-onset sepsis versus controls per time period in Validation Cohort 2.** Bacterial β-diversity as assessed by PCoA based on Bray-Curtis dissimilarity is displayed. Potential clustering of fecal samples based on microbiome composition is assessed for all infants per time period (-10 to -9, -8 to -7, -6 to -4, and -3 to -1 days prior to LOS onset) in **A)** infants with LOS versus controls, **B)** infants with *S. aureus*-LOS, non-staphylococcal LOS, and controls, and **C)** *E. coli*-LOS versus controls. Only one sample per infant (closest to LOS) per time period was analyzed to account for repeated measures. Statistical analysis was performed by PERMANOVA. A p-value below 0.05 was considered significant.

**
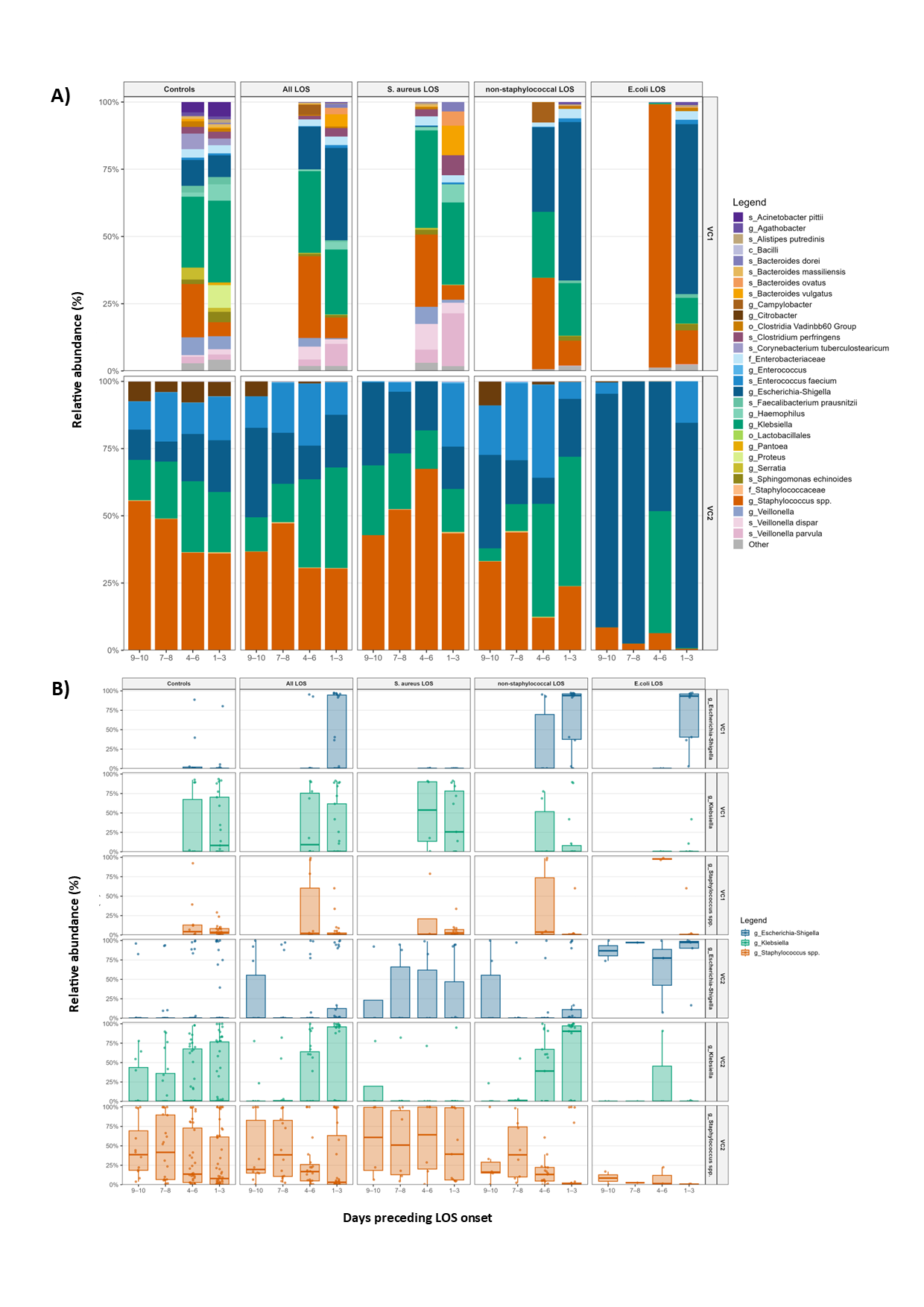
**

**Figure S14. Temporal dynamics of gut microbiota composition at the genus level during the ten-day window preceding clinical onset of late-onset sepsis (LOS) in infants and controls in Validation Cohort (VC) 1 and 2. A)** The figure presents stacked bar charts illustrating the relative abundance (Y-axis, %) of the 25 predominant bacterial genera across the absolute time points prior to LOS onset or the corresponding t=0 of the control infants. From left to right, panels depict the microbial composition of control infants, infants with LOS caused by any pathogen (All LOS), infants with *Staphylococcus aureus* LOS, infants with non-Staphylococcal LOS, and infants with *Escherichia coli* LOS. **B)** Sample-level relative abundance (%) of g_*Escherichia–Shigella*, g_*Klebsiella*, and g_*Staphylococcus* spp. across four time intervals preceding late-onset sepsis or the corresponding t=0 of control infants in VC1 and VC2. Boxplots show median and interquartile range (Q1–Q3); points indicate individual samples. Panels depict Controls, all LOS, *S. aureus*-LOS, non-staphylococcal LOS, and *E. coli*-LOS.


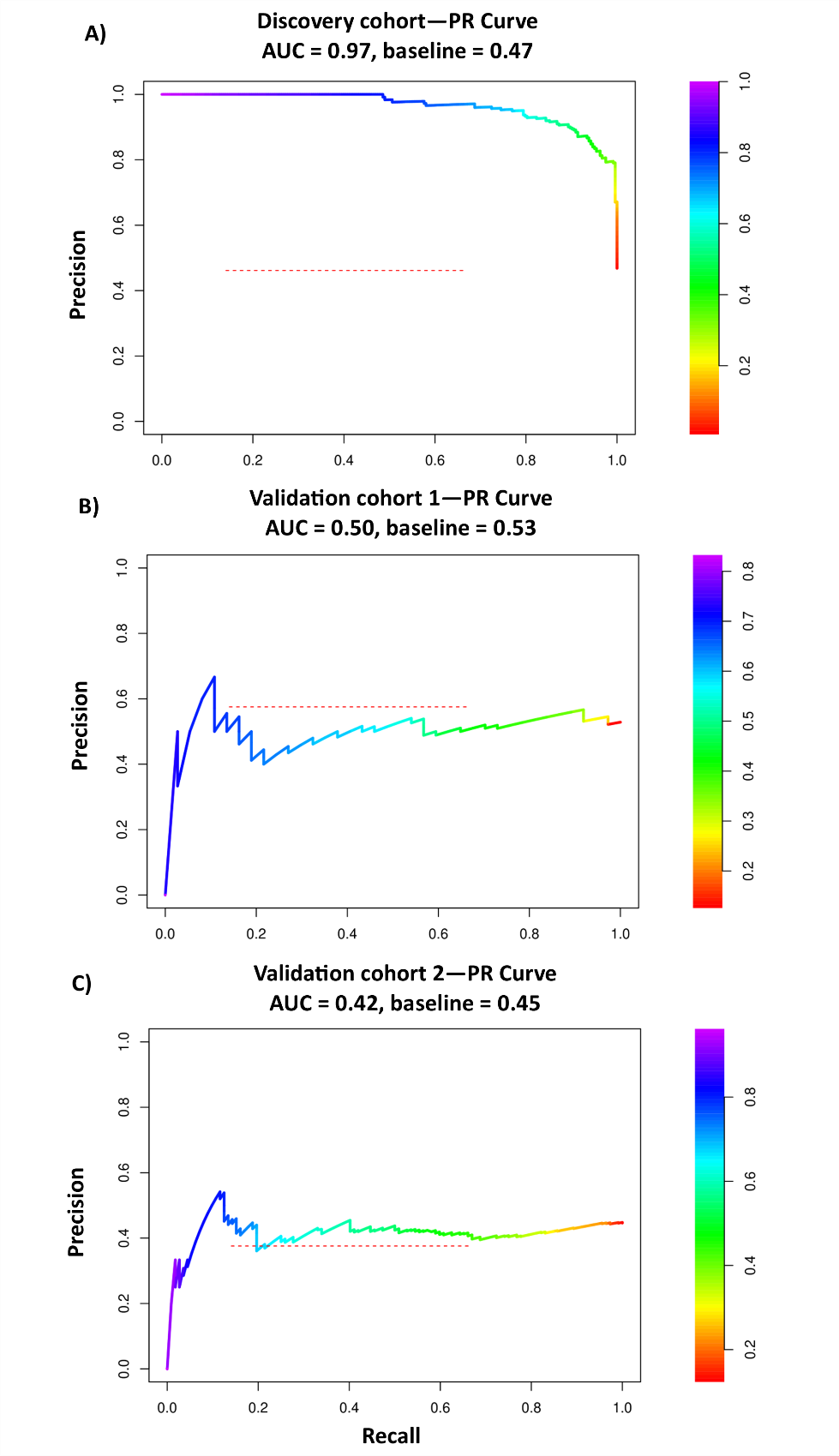


**Figure S15. PR-AUC curve of non-staphylococcal LOS model across cohorts.** The PR curve plots precision (positive predictive value) against recall (true positive rate) across varying classification thresholds. The PR-AUC quantifies the model's ability to correctly identify positive cases, with the dashed horizontal line representing the no-skill baseline defined as the prevalence of the positive class in the dataset. Higher PR-AUC values relative to the baseline indicate better discriminatory performance, particularly for the minority class. Panel **A** displays the PR curve in the Discovery Cohort, **B** in Validation Cohort 1, and **C** in Validation Cohort 2. *Abbreviations: AUC = Area Under the Curve, PR: precision-recall, LOS: late-onset sepsis.*


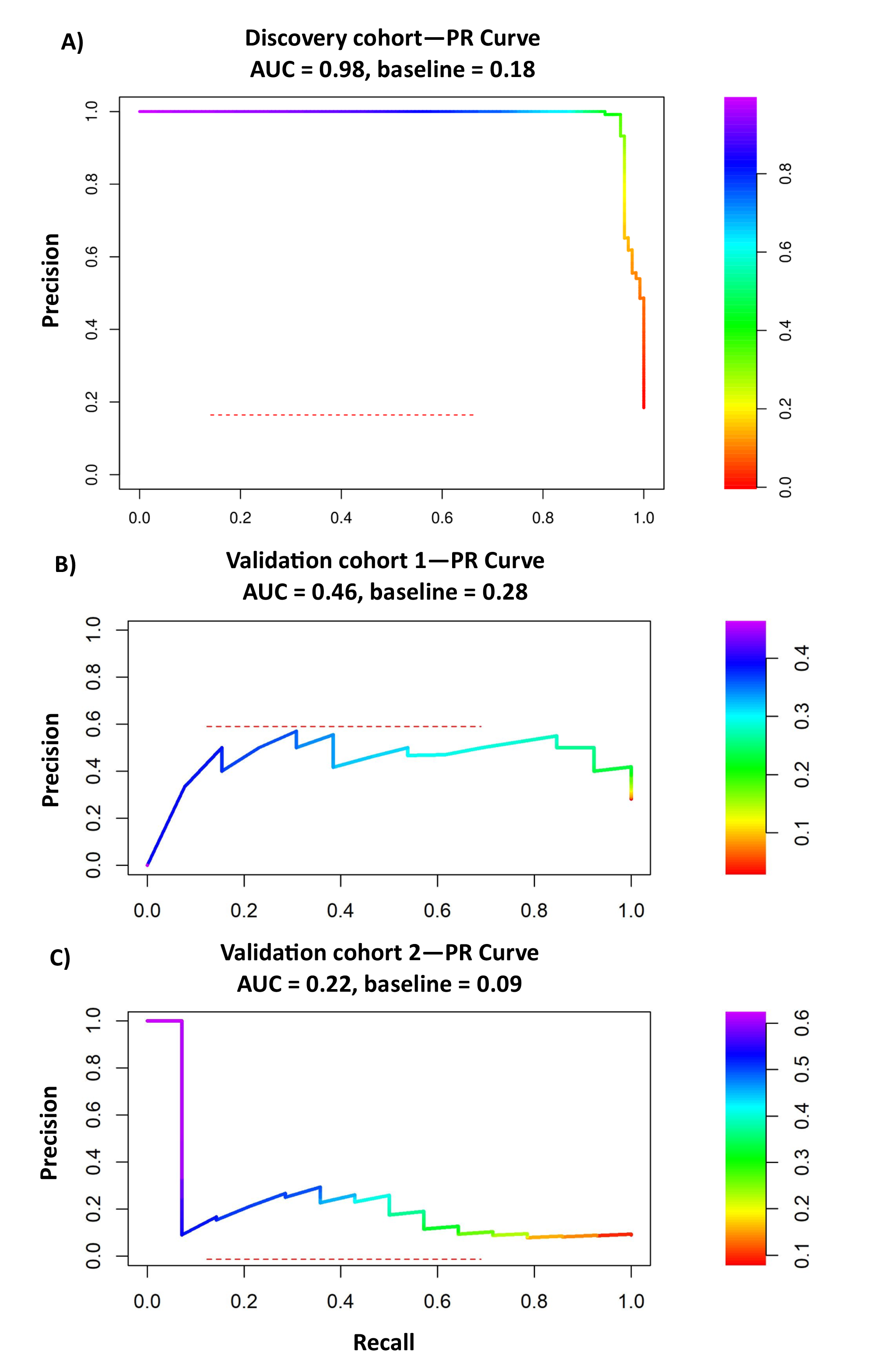


**Figure S16. PR-AUC curve of the *Escherichia coli* LOS model across cohorts.** The PR curve plots precision (positive predictive value) against recall (true positive rate) across varying classification thresholds. The PR-AUC quantifies the model's ability to correctly identify positive cases, with the dashed horizontal line representing the no-skill baseline defined as the prevalence of the positive class in the dataset. Higher PR-AUC values relative to the baseline indicate better discriminatory performance, particularly for the minority class. Panel **A** displays the PR curve in the Discovery Cohort, **B** in Validation Cohort 1, and **C** in Validation Cohort 2. *Abbreviations: AUC = Area Under the Curve, PR: precision-recall, LOS: late-onset sepsis.*
